# Metasurface platform for simultaneous and uncorrelated emissivity control over MWIR and LWIR spectral bands

**DOI:** 10.1038/s41377-026-02426-y

**Published:** 2026-07-23

**Authors:** Roy Maman, Noa Mazurski, Ilya Goykhman, Uriel Levy

**Affiliations:** Institute of Applied Physics, The Faculty of Science, The Center for Nanoscience and Nanotechnology, The Hebrew University of Jerusalem, Jerusalem, 91904 Israel

**Keywords:** Mid-infrared photonics, Metamaterials, Imaging and sensing, Nanophotonics and plasmonics, Optoelectronic devices and components

## Abstract

Thermal radiation in the mid-wave infrared (MWIR) and long-wave infrared (LWIR) underpins a wide range of imaging, sensing, and security technologies. While metasurfaces have enabled spectral and angular control of thermal emissivity, achieving spatially resolved and uncorrelated emissivity control across multiple infrared bands on a single platform remains a major challenge. Here we experimentally demonstrate a metasurface-based approach that enables pixel-level control of thermal emissivity in both the MWIR and LWIR bands simultaneously. The platform is based on a cavity-coupled metal-insulator-metal architecture supporting two infrared resonances whose spectral positions and strengths are orthogonally controlled through geometric design parameters. By systematically exploring the design space, we realize a broad emissivity palette and experimentally verify its thermal response using Fourier-transform infrared spectroscopy (FTIR) together with calibrated MWIR and LWIR thermal imaging. This capability allows the encoding of spatial thermal patterns that are either correlated or distinct in the two bands, enabling demonstrations of dual-band thermal camouflage and decoupled thermal image multiplexing on a single chip. The metasurface response is shown to be polarization-insensitive and robust over a wide range of temperatures and viewing angles. These results establish a scalable route toward multi-band, image-level control of thermal emission, with potential applications in thermal imaging, security, and infrared information encoding.

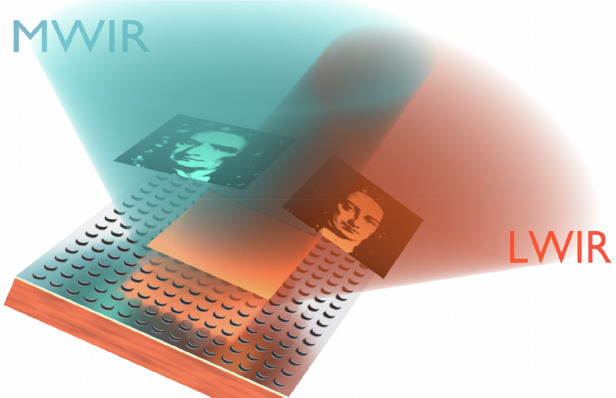

## Abstract (web summary)

"A cavity-coupled metasurface platform achieves independent, dual-band grayscale emissivity control, enabling simultaneous thermal camouflage and decoupled multi-channel image multiplexing across the mid- and long-wave infrared spectral windows."

## Introduction

Thermal emission within the mid-wave infrared (MWIR, 3–5 μm) and long-wave infrared (LWIR, 8–14 μm) wavelength ranges plays a central role in modern infrared imaging, sensing, and security systems. These two atmospheric windows are central to modern thermal detection systems, which increasingly operate in both bands to enhance contrast, robustness, and information content. Consequently, the ability to control thermal emission spectrally and spatially across MWIR and LWIR has become a key objective in thermal photonics.

In recent years, engineered photonic structures have emerged as a powerful platform for manipulating thermal emissivity. By tailoring optical resonances, metasurfaces enable control over the spectral, angular, and polarization properties of thermal radiation^1–15^. Such approaches complement thermal manipulation through engineered heat conduction^16,17^, which can regulate total emitted power but is fundamentally limited by Fourier’s law of thermal diffusion and does not enable localized, pixel-level control. As a result, photonic control of emissivity has become central to applications such as thermal camouflage, radiative cooling, information encryption, and anticounterfeiting^18–30^.

Modern thermal detection typically relies on MWIR photon detectors and LWIR thermal bolometers, motivating device concepts that operate across both spectral bands. Existing approaches address multispectral operation either by stacking multiple functional layers^31–34^ or by employing metasurfaces supporting multiple resonances^35^. However, most demonstrated devices provide spatially uniform emissivity, limited grayscale control, or strongly correlated responses between spectral bands. While adaptive control has been explored using phase-change materials^35–43^, liquid crystals^2,44^, or graphene integration^45–47^, achieving simultaneous, spatially resolved, and spectrally selective emissivity control in both MWIR and LWIR on a single planar platform remains a major challenge. A comparative overview of representative platforms and their performance metrics is provided in Table 1.

**Table 1:** Horizontal comparison of dual-band thermal emission platforms

Platform	Spectral decoupling	Band control	Pixel size (µm)	Angular stability	Temperature stability	Dual-band image multiplexing
Lithography-free multilayer^31^	None	Correlated dual-band (binary)	—	Wide-angle	Up to 300 °C	No
Multi-resonance metasurface^27,35^	NA	Single-band tuning	10-100	Limited	Not reported	No
Multilayer metasurfaces^28,30,32–34^	Correlated	Binary dual-band	10–1000	Work-dependent	Moderate range	No
**This work**	**Uncorrelated**	**Simultaneous MWIR** + **LWIR control**	**~200**	**LWIR: 60°; MWIR** < **35°**	**39–112** **°C (experimental)**	**Yes**

Bold values indicate the results of this work, presented for comparison with previously reported platforms

This limitation is particularly critical for thermal camouflage and thermal encoding. In camouflage applications, the goal is to suppress or tailor the thermal signature of an object so that it blends into its background, which generally requires grayscale emissivity control in both MWIR and LWIR, robustness to polarization and viewing angle, and stability over a wide temperature range. In contrast, thermal encoding aims to generate structured thermal signals that can be detected and interpreted using standard thermal imaging systems. Despite their opposing objectives, thermal camouflage and thermal encoding can be viewed as reciprocal problems that rely on the same underlying capability: precise, pixel-level control of thermal emissivity.

Here we experimentally demonstrate, for the first time, a metasurface platform that enables uncorrelated and simultaneous spatial control of emissivity in both the MWIR and LWIR bands. Our cavity-coupled metal–insulator–metal design supports two infrared resonances whose spectral positions and strengths are effectively decoupled through geometric parameters, enabling a broad emissivity palette. Using this platform, we realize dual-band thermal camouflage as well as uncorrelated thermal image multiplexing on a single chip. The metasurface response is polarization-insensitive, robust over a wide range of viewing angles and temperatures, and compatible with large-scale fabrication, providing a scalable route toward image-level control of thermal emission.

To quantify thermal emission in each spectral band, we consider the radiative power emitted by a body in thermal equilibrium. The emitted radiation power within a spectral band is given by Planck’s law integrated over the wavelength range:

1 $$P\left(T\right)=A\mathop{\int}\limits^{{\lambda }_{2}}_{{\lambda }_{1}}{\epsilon }_{\lambda }\frac{2\pi h{c}^{2}{\lambda }^{-5}}{{e}^{\displaystyle\frac{{hc}}{\lambda {k}_{B}T}}-1}d\lambda$$

where $A$ is the emitting area, $T$ is the body’s temperature, ${\lambda }_{\mathrm{1,2}}$ define the spectral band, and *ϵ*_*λ*_ is the spectral emissivity. In MWIR photon-detection systems, the detected signal is proportional to the number of emitted photons, given by

2 $${N}_{{photons}}(T)=A\mathop{\int}\limits^{{\lambda }_{2}}_{{\lambda }_{1}}{\epsilon }_{\lambda }\frac{2\pi c{\lambda }^{-4}}{{e}^{\displaystyle \frac{{hc}}{\lambda {k}_{B}T}}-1}d\lambda$$

According to Kirchhoff’s law of thermal radiation, under Lorentz reciprocity, the spectral emissivity,$\,{\epsilon }_{\lambda }$, equals the spectral absorptivity of the object^48,49^. Therefore, by engineering the absorptivity of a photonic structure, it is possible to directly tailor its thermal emission in both MWIR and LWIR bands.

## Results

**Fig. 1:**
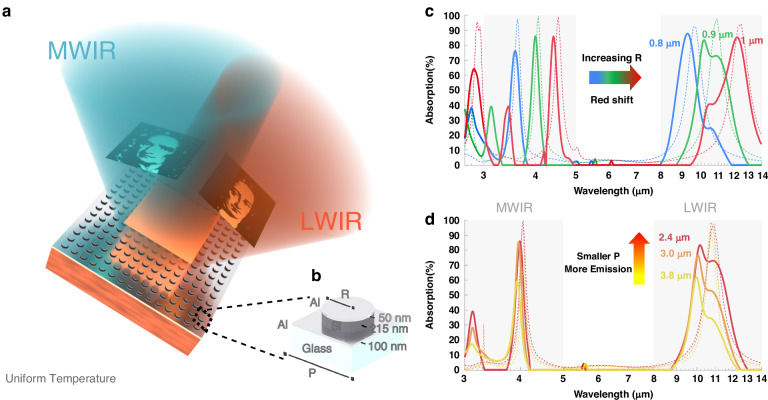
Device concept and geometry-dependent MWIR–LWIR metasurface absorption. **a** A sketch showing both MWIR and LWIR emission from a heated metasurface and the corresponding infrared images. **b** an illustration of a metasurface unit cell. The resonances are created by an MIM structure. The substrate is coated with a 100 nm thick layer of Al and two disk-shaped layers (215 nm amorphous silicon, 50 nm Al). The radius (R) and period (P) of the disk control the absorption spectrum, according to Fig.1c, d. The periodic array size is $200x200\mu {m}^{2}$. **c** FTIR-measured (solid lines) and FDTD-simulated (dashed lines) absorption spectra for three metasurfaces with the same period ($P=2.4\mu m)$ but different radius ($R=\mathrm{0.8,0.9,1}\mu m)$. Both MWIR and LWIR absorption resonances are noticeable. Increasing the radius of the unit cell inflicts a red shift for both resonances. **d** FTIR-measured (solid lines) and FDTD-simulated (dashed lines) absorption spectra for three metasurfaces with identical disk radius (R = 0.9 μm) and different periods (*P* = 2.4, 3.0, and 3.8 μm). Increasing the unit-cell period results in a reduced spectral absorption

A schematic of the cavity-coupled plasmonic metasurface structure that is used to control emissivity is shown in Fig.1. The concept is sketched in Fig. 1a, where the metasurface controls the emission of radiation in the MWIR and the LWIR spectral regime. Design flexibility allows the generation of either identical images or very different images at these two spectral bands. The device is a metal-insulator-metal (MIM) metasurface on top of a bulk substrate. The metasurface consists of a thin uniform metallic layer, with an array of insulator-metal disks on top of it. The structure is symmetric for $\frac{\pi }{2}$ rotations and is therefore polarization insensitive under normal incident angles. A unit cell of this metasurface is shown in Fig. 1b. For disks’ radii of $0.7-1.2\mu m$, the array has two relevant cavity-mode resonances, one in the MWIR band and one in the LWIR band. At resonance, the trapped photons excite surface plasmons whose energy dissipates by ohmic loss^27,50^. The spectral absorption spectrum (which according to Kirchhoff’s law is equivalent to the spectral emissivity) is measured with an FTIR (Bruker’s HYPERION II FTIR microscope). Increasing the radius of the disk increases cavity’s length and therefore red shifts both resonances (Fig.1c). A second degree of freedom for controlling the emissivity is the unit-cell period. Each pixel in the device occupies a finite area and is composed of a periodic repetition of the unit cell. Pixels with larger unit-cell periods contain fewer resonant elements overall, which degrades the effective periodicity of the structure. As a result, the resonant response is weakened compared with simulations assuming an infinitely periodic metasurface (see Supplementary Note 1). This property of the design enables scaling of the absorption amplitude with minimal modification of the spectral response. Overall, the total absorption of the pixel is controlled by the unit-cell radius (R) and period (P), enabling orthogonal control over the resonance wavelength and absorption strength.

In modern thermal imaging technology, LWIR detection is dominated by uncooled thermal bolometers in which the signal is proportional to radiation power, while MWIR detection is dominated by cooled photon detectors in which the signal is proportional to the number of photons. Thus, to calculate the expected thermal emission of a structure for a given temperature, we extracted the spectral absorption values from the simulation and plugged them in eq.1 (LWIR band) and Eq.2 (MWIR band). The outcome was then divided by blackbody radiation (calculated using the same equation, assuming spectral emissivity of ${\epsilon }_{\lambda }=1$ over the entire band) to extract the relative thermal emission in both bands. This relative thermal emission served as a figure of merit in the exploration of the parameter space spanned by the radius and period of the unit cell presented earlier. Structures with a radius value that creates resonance in both MWIR and LWIR bands ($0.7-1.2\mu m)$ were simulated using FDTD solver for various feasible periods with the goal of creating a broad emissivity palette, allowing the uncorrelated representation of thermal signals in both MWIR and LWIR bands simultaneously. This effective decoupling is enabled by the strong spatial confinement of the cavity modes, which prevents the MWIR and LWIR fields from mixing. To strictly quantify this independence and exclude regions of nonlinear coupling, we performed a global Jacobian sensitivity analysis (see Supplementary Note 4 for physical mode analysis and stability mapping).

**Fig. 2:**
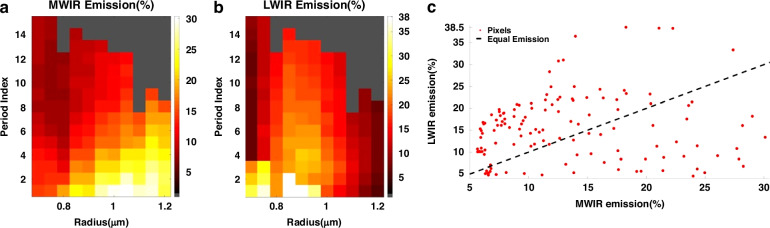
The relative emission of 130 different structures. The relative thermal emission in MWIR (**a**) and LWIR bands (**b**) side by side, as extracted from thermal cameras and normalized using a blackbody. Structures with different periods (total of 15 periods ranging from $2R+0.2\mu m$ to $5.2\mu m$) were measured for every radius between 0.7 to 1.2$\mu m$. **c** Relative LWIR emission Vs. relative MWIR emission of the measured structures

Overall, 130 different structures were fabricated and measured using FTIR microscope. Measurement results are presented together with simulation references in Supplementary Note 2 Fig. S3. Each structure consists of a $200x200\mu {m}^{2}$ periodic lattice representing a unique combination of period and radius. Figure 2a shows the relative thermal emission of the different structures, as extracted from thermal images captured independently with a cooled MWIR camera (FLIR Neutrino LC) and an uncooled LWIR camera (FLIR Boson). In both images, the structures were uniformly heated to $54^\circ c$. In Fig. 2c, the relative thermal emission of all structures is represented in a scattered plot, presenting the relative LWIR emission of each structure against its relative MWIR emission. Across all measured structures, MWIR and LWIR emissivities show negligible correlation, with neither linear (Pearson $R=0.10,{p}=0.26$) nor monotonic (Spearman $\rho =0.14,{p}=0.11$) dependence, indicating uncorrelated spectral control. For a perfect decoupled device, this scatter plot would have independent values ranging from 0% to 100% in each axis so that for every relative MWIR emission and for every relative LWIR emission there would be plethora of available structures to choose from. Our measured structures provide up to 38.5% relative LWIR emission and 30% relative MWIR emission, with better coverage in values under 25% for both bands (using bigger structures, we can get peak relative emissions of 44.3% in the LWIR and 33.7% in the MWIR. Supplementary Note 1).

**Fig. 3:**
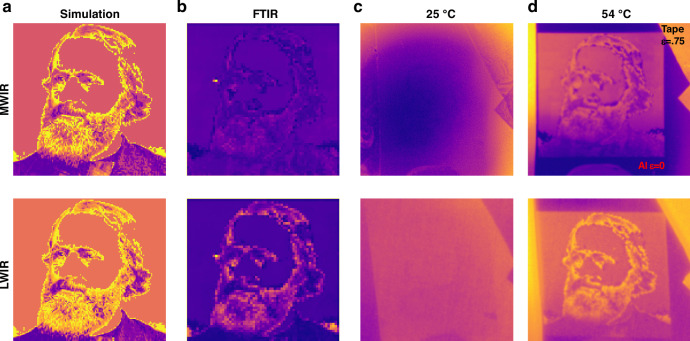
Simultaneous thermal encoding in both MWIR and LWIR on a single chip. **a** Simulation of the thermal image on a uniform background. **b** The expected thermal emission based on the FTIR measurements of the chip. **c** The thermal images of the chip at room temperature. **d** The thermal images of the chip heated to$54^{\circ}{c}$

To demonstrate the usefulness of our platform for thermal camouflage, we have used our previously characterized 130 structures to form an image on a substrate having a uniform temperature. We created a neutral background with fixed thermal emissions in both ( ~ 17% for LWIR and ~13% for MWIR) bands by embedding the same chosen structure in the background areas. Then, we embedded different structures to create the elaborated outline of Gustav Kirchhoff as an image, where each structure represents a pixel in both the MWIR and the LWIR image. By choosing pixels having similar thermal emissivity in both bands, we were able to obtain the same apparent thermal image in MWIR and in LWIR. Figure 3 shows the experimental results in both bands simultaneously on the same chip. The left column displays simulated images of Kirchhoff on a flat background. Each pixel in the simulation corresponds to one of the 130 structures as measured before. Next, the simulated pixels were fabricated, and their absorption values were measured by FTIR. The second column displays the expected emission in both bands based on eq.1 (LWIR) and Eq. 2 (MWIR) using the measured spectral absorption to represent the emissivity according to Kirchhoff’s law. A thermal pixel with emissivity $\epsilon$ would also have a complementary reflectivity of $1-\epsilon$. The total contribution of the pixel to the thermal signal consists of its self-emission and reflections from the environment. Around room temperature, the emission of the pixel blends with the thermal emission of the environment so the image disappears in the background (see third column). At higher temperatures, self-emission of the pixel is becoming the dominant source of the signal. The right column of Fig. 3 shows the thermal image from the chip at temperature of $54^\circ c$. The thermal image, in both LWIR and MWIR shows the image on a flat background, surrounded by a zero-emissivity Al coating and a polyimide tape ($\epsilon =0.75$) used for thermal conductivity.

**Fig. 4:**
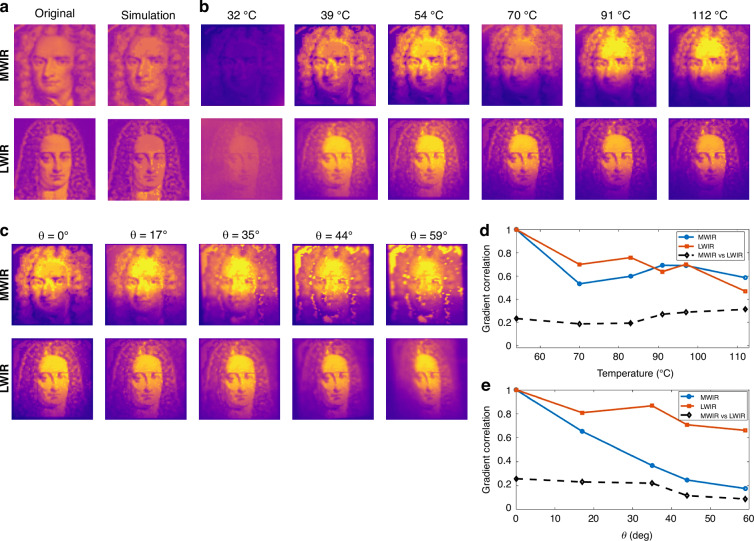
Uncorrelated thermal encoding in both MWIR and LWIR on a single chip. Images of Newton and Leibniz are thermally encoded on MWIR and LWIR respectively. Each pixel is implemented using one of the 130 characterized metasurface structures. **a** Original target images (left) and simulated thermal emission based on selected emissivity values (right) for both MWIR and LWIR channels. **b** Experimentally measured thermal images at different chip temperatures (32 °C–112 °C). As temperature increases, the encoded patterns become dominant over the background emission. **c** Thermal images acquired at 54 °C for different incident angles (0°, 17°, 35°, 44°, and 59°). **d** Gradient Correlation as a function of temperature. for each spectral channel, metrics are calculated relative to the 54 °C reference image to quantify thermal robustness. Cross-band (MWIR–LWIR) correlations are shown head-to-head to evaluate crosstalk suppression. All sampled images are shown in Supplementary Note 3.**e** Gradient Correlation as a function of incident angle. For angular robustness, each channel is evaluated relative to the 0° baseline, while cross-band (MWIR–LWIR) correlations directly quantify spectral decoupling. The LWIR channel maintains high fidelity across the full angular range, whereas the MWIR channel exhibits degradation over sampled range

The ability to spatially control the emissivity with gray-level selectivity, in both MWIR and LWIR simultaneously is very appealing for thermal camouflage, as for most objects, the thermal signatures in both bands are highly corelated (see Supplementary Note 3.3). For general thermal encoding applications, one seeks effectively decoupled control over the two bands. To demonstrate this ability, we encoded two different images (Newton and Leibniz) on the same chip – one in the MWIR band and another one in the LWIR band. Figure 4a shows the original images to be encoded (left) and the simulation of the image based on the emissivity values that were chosen (right). Minor differences between the original and simulated images arise from the finite coverage of the MWIR–LWIR emissivity space (Fig. 2b and Supplementary Note 4). Figure 4b shows the experimental results of the chip at different temperatures. The chip was imaged by thermal cameras under different temperatures. As in the previous case, at room temperature the thermal signal from the encoded image cannot be separated from the background signal. At $32^\circ c$ the images start to appear, yet they both look faded. As the chip temperature increases, the thermal images become more prominent. The device was experimentally characterized over a temperature range from 25°C to 112°C, throughout which the MWIR and LWIR images remained distinct and stable. For temperatures above 54°C, MWIR images were acquired using a neutral density filter (OD 1.0) to prevent detector saturation. The observed thermal stability is influenced by a combination of systemic radiometric effects (governed by Wien’s displacement law) and material-specific properties - most notably the thermo-optic effect (${dn}/{dT}$) in the amorphous silicon spacer (see Supplementary Note 3). The structural upper thermal limit is determined by potential inter-diffusion or oxidative stress at the metal–dielectric interfaces, expected to exceed 300 °C.

To quantitatively evaluate multiplexing performance and crosstalk suppression, we analyzed the Gradient Correlation (GC) across the full temperature range. The cross-band GC values (Fig. 4d) remain consistently low ( ≈ 0.2) up to 112 °C, confirming strong suppression of high-frequency feature leakage between MWIR and LWIR channels.

The angular robustness of the multiplexed encoding was evaluated up to 59° (Fig. 4c). The encoded signatures remain clearly identifiable across the tested range. Quantitative analysis of GC versus incident angle (Fig. 4e) shows that the LWIR channel maintains high fidelity throughout the 0°–59° range, while the MWIR channel to degrades linearly.

Notably, these metrics are conservative estimates, as they are sensitive to experimental artifacts such as slight variations in camera focus, saturation, pixel registration, and sample orientation across different measurement sessions; while these are partially compensated for during post-processing, the intrinsic performance of the metasurface is expected to exceed the values reported here. Together, these measurements define the practical temperature and angular capacity limits of the dual-band multiplexing platform.

## Discussion

This paper demonstrates numerically and experimentally a metasurface platform capable of effectively uncorrelated and simultaneous spatial control of emissivity in both the MWIR and LWIR bands. As such, it represents a significant step forward in the long-lasting effort to provide more flexibility in thermal imaging scenarios. Our approach enables a broad emissivity palette, sufficient for constructing complex thermal patterns in both bands on the same chip.

On top of operating in both spectral bands, another key outcome of our work is the realization of thermal images that remain robust over a wide range of viewing angles and temperatures. The demonstrated stability up to ±35° angle of incidence and across a broad thermal range confirms that the platform is suitable for plethora of realistic scenarios, where environmental and operational conditions cannot be precisely controlled. The use of a simple MIM structure further underlines the practicality of the approach. Moreover, while the samples were fabricated using E-beam lithography, we have also shown the feasibility to fabricate such structures using conventional photolithography, paving the way for scaling to large-area devices, an essential requirement for camouflage and security applications.

The relative emissions achieved (up to ~38.5% in LWIR and ~30% in MWIR) demonstrate the feasibility of grayscale emissivity control. Most previously reported thermal metasurfaces operate primarily in a binary emissivity regime^28,30–34^, switching between high and low emission states, thereby maximizing contrast but limiting grayscale control. Multi-resonant metasurface platforms have demonstrated single band grayscale control^27,35^. Quantitative emissivity values were not provided but the reported performance appears comparable in magnitude to the effective emissivities achieved here. Importantly, although the maximum effective emissivities are moderate compared to ideal blackbody values, their practical radiometric impact is substantial due to the nonlinear temperature–radiance relation. As shown in Supplementary Note 5, emissivity values below 0.4 still produce substantial apparent temperature shifts; for instance, under a 20 °C background, a 100 °C object with ε = 0.4 appears at ~60 °C. A quantitative analysis of environmental adaptability and radiometric contrast under varying background temperatures is provided in Supplementary Note 5.

Physically, this relative emissivity range is inherently bounded by the narrowband nature of localized MIM resonances compared to the broad atmospheric windows. Future designs could significantly expand this dynamic range by employing multiplexed super-cells, incorporating multiple disk radii per pixel, to broaden the absorption bandwidth. However, such multiplexing introduces a geometric constraint: fitting multiple resonators into a compact area requires smaller disk radii, which inherently blue-shifts the resonances out of the target thermal windows. To counteract this, future architectures could locally vary the dielectric spacer thickness, utilizing the cavity’s RLC dependence to red-shift the resonances back into the desired bands without needing to expand the pixel footprint (Supplementary Note 3.1). Furthermore, pushing spatial resolution below our current 200 μm pixel size introduces a fundamental trade-off: higher perimeter-to-area ratios increase edge scattering and degrade collective radiative coupling, reducing overall emission (Supplementary Note 1). To reach the ~15 μm scale of commercial sensors without sacrificing emission strength, boundary-matching guard rings could be integrated to suppress these finite-array edge effects. Crucially, because the inter-resonator period serves as the primary independent degree of freedom for scaling emission amplitude, any such advanced multidimensional architectures must carefully preserve this geometric tunability to maintain the platform’s signature uncorrelated grayscale control.

Our emissivity palette is not yet fully continuous. Nevertheless, we observed only minor discrepancies between the target images and the realized thermal patterns. Expanding the design space, through alternative geometries or additional resonant modes, could enrich the accessible emissivity states and improve the fidelity of thermal encoding.

The demonstrated capability of encoding different images in MWIR and LWIR simultaneously illustrates the versatility of the platform. While camouflage applications typically demand correlated emissivity in both bands, thermal information encoding benefits from spectral independence. Our results show that it is possible to encode two distinct patterns, opening opportunities for dual-band multiplexing, anticounterfeiting, and secure communication^18–20,23^.

Looking ahead, dynamic control represents a natural extension of this work. Incorporating phase-change materials^38–41^, electro-optic layers^2,44^, or graphene^22^ could enable real-time reconfiguration of emissivity states, adding adaptability to the demonstrated static encoding platform. Coupling our design with advanced computational inverse design strategies could further optimize the palette coverage and tailor device response to specific operational requirements.

In summary, this work establishes a metasurface-based approach for dual-band thermal emission control that is scalable, stable, and versatile. By addressing both camouflage and encoding requirements, this work highlights how pixel-level emissivity control can support emerging applications in security, communication, and thermal management.

## Materials and methods

### Simulations

The dual resonance MIM structures were simulated in an FDTD solver.

The spectral emissivity of each structure was assumed to be equal to the spectral absorption (ϵ_λ_=1-R-T) according to Kirchhoff’s law. The expected thermal emission was calculated using Planck’s law (eq.1 for LWIR and Eq. 2 for MWIR) with the simulated spectral emissivity values. The blackbody emission was calculated too according to Planck’s law with $\epsilon =1$ for all wavelengths. The relative emission of a structure is its expected thermal emission divided by the blackbody emission for the same temperature. Different structures were simulated with the goal of modulating the resonances in MWIR and LWIR to gain various relative emission values in both bands, while considering fabrication tolerances. The unit cell is rotationally symmetric to maintain polarization insensitivity, but not all possible unit cells were simulated. The palette of emissivity values may be expended by exploring more structures. Polarization dependent structures can be created using elliptical structures^13,14^.

### Fabrication

The metasurface consists of a metal–insulator–metal (MIM) stack composed of a 100 nm Al ground plane, a 215 nm amorphous silicon (a-Si) layer, and patterned 50 nm Al disks forming the top resonators. A 100 nm aluminum layer was first deposited onto Borofloat 33 glass substrates using electron-beam evaporation (Angstrom Quantum Series). Subsequently, a 215 nm amorphous silicon layer was deposited by plasma-enhanced chemical vapor deposition (PECVD, Oxford PlasmaLab System 100). The a-Si thickness was verified by spectroscopic ellipsometry. For the devices reported in the main text, patterns were defined by electron-beam lithography (EBL) using PMMA resist. After resist development, a 50 nm Al layer was deposited by electron-beam evaporation followed by lift-off in Acetone. The Al thickness was verified using a profilometer (Dektak XT, Bruker) on calibration patterns. The last step was etching of the a-Si using the 50 nm Al as a hard mask, transferring the Al pattern to the a-Si layer. The Al disk thickness remained unchanged during the etching process. To assess scalability, identical geometries were fabricated using standard photolithography with a calibration mask. SEM characterization confirmed comparable disk morphology, diameter uniformity, and periodicity between EBL and photolithography-fabricated structures (Supplementary Note 2, Fig. S5).

### Emissivity measurements

We measured the emissivity of the devices using Bruker’s HYPERION II FTIR microscope in reflection mode, assuming zero transmission and based on Kirchhoff’s law. We integrated over the FTIR-measured spectral absorption to calculate the MWIR and LWIR expected emissions and verified the results using a blackbody, as elaborated in the optical measurements section.

### Optical measurements

The LWIR images were sampled with uncooled Teledyne FLIR Boson camera (pixel size 12$\mu m)$, equipped with a18 mm camera lens, together with an f = 50 mm coated ZnSe plano-convex lens (Thorlabs LA7656-E2) resulting in an effective pixel size (pixel size on the sample) of $\sim 33\mu m$. The MWIR images were sampled with cooled Teledyne FLIR Neutrino camera (pixel size 15$\mu m)$, using a coated ZnSe achromatic doublet (Thorlabs AC254-200-E, f = 200 mm) together with an f = 50 mm coated ZnSe aspheric lens (Thorlabs AL72550-E) resulting in an effective pixel size of $\sim 60\mu m$. For measurements above 54 °C, the MWIR signal approached detector saturation. To maintain operation within the linear dynamic range of the camera, a neutral density filter (Thorlabs NDIR10A, OD = 1) was inserted in the MWIR optical path. The sample was heated using a resistor taped to it with a conductive polyimide tape. The sample’s temperature was measured using a thermocouple. To measure the relative emissions of the device, we calibrated the thermal cameras using a blackbody (CI Systems SR800N-4A) set to the same temperature as the measured temperature of the sample.

### Metrics

To quantitatively assess the fidelity of the multiplexed thermal images and the efficiency of channel decoupling, two primary metrics were employed: (SSIM and Gradient Correlation.

SSIM is used to measure the perceived change in structural information between the experimental thermal images.

3 $${SSIM}\left(x,y\right)=\frac{{(2\mu }_{x}{\mu }_{y}\,+\,{C}_{1})(2{\sigma }_{{xy}}\,+\,{C}_{2})}{{(\mu }_{x}^{2}+{\mu }_{y}^{2}+{C}_{1})({\sigma }_{x}^{2}+{\sigma }_{y}^{2}+{C}_{2})}$$

where $\mu$ and $\sigma$ represent the mean and variance of the intensity distributions, and C are constants to ensure stability at low denominators. SSIM is particularly sensitive to the global thermal footprint and macro-scale geometry of the chip.

To specifically evaluate the suppression of crosstalk between the MWIR and LWIR channels, we utilized Gradient Correlation (GC). This metric focuses on high-frequency spatial details (edges and textures) by calculating the correlation between the gradient maps of the images:

4 $${GC}=\frac{{\sum }_{i.j}\nabla {x}_{i,j}\cdot \nabla {y}_{i,j}}{\sqrt{{\sum }_{i.j}{\left|\nabla {x}_{i,j}\right|}^{2}\cdot {\sum }_{i.j}{\left|\nabla {y}_{i,j}\right|}^{2}}}$$

where $\nabla x$ and $\nabla y$ are the spatial gradients of the MWIR and LWIR images, respectively. Because the encoded patterns (e.g., the distinct portraits) are defined by their sharp emissivity transitions, GC serves as a more rigorous indicator of information decoupling. A low cross-band GC confirms that the high-frequency information encoded in one band does not “bleed” into the other, even if the macro-scale thermal signatures overlap.

## Supplementary information

SUPPLEMENTAL MATERIAL

## Data Availability

The datasets generated and analyzed during this study are available from the corresponding author upon reasonable request.
