# Supplementary material for: Metasurface platform for simultaneous and uncorrelated emissivity control over MWIR and LWIR spectral bands
Source: Light Sci Appl. 2026 Jul 23;15:329. doi: 10.1038/s41377-026-02426-y (PMC13396640; doi:10.1038/s41377-026-02426-y)
Supplement: Supplementary file 1 — SUPPLEMENTAL MATERIAL [file 41377_2026_2426_MOESM1_ESM.docx]

**Supplementary Information for Metasurface platform for simultaneous and uncorrelated emissivity control over MWIR and LWIR spectral bands**

*Roy Maman*, Noa Mazurski, Ilya Goykhman and Uriel Levy*

Institute of Applied Physics, The Faculty of Science, The Center for Nanoscience and Nanotechnology, The Hebrew University of Jerusalem, Jerusalem 91904, Israel

E-mail: [Roy.maman@mail.huji.ac.il](mailto:Roy.maman@mail.huji.ac.il).

**1 - SUPPLEMENTARY NOTE: Impact of Finite Pixel Size and Unit-cell Period on Absorption**

For a given choice of layer thickness, the two primary geometrical parameters governing the response of the metasurface are the unit-cell radius and period. Varying the unit-cell radius induces a pronounced spectral shift of the resonant response, whereas simulations of infinite periodic structures indicate that changes in the period have only a minor influence on the resonance wavelength (see Fig. 1d of the main manuscript), indicating that the coupling between adjacent resonators is weak. In contrast, measurements performed on fabricated devices reveal a uniform reduction in the overall absorption for structures with larger unit-cell periods. This discrepancy arises from the finite size of the metasurface arrays in the experimental devices.

To achieve well-defined resonances in both the MWIR and LWIR bands, the relevant unit-cell radius varies in the range of 0.7-1.2$\mu m$. For a unit cell with radius *R*, the smallest possible period is 2*R*. Consequently, the smallest period common for all relevant radii is 2.4 $\mu m$, while the largest tested period was 5.2$\mu m$. Within this parameter space, we fabricated and characterized 130 distinct metasurface designs spanning different combinations of radii and periods, measured using FTIR spectroscopy (see Supplementary Note 2 for details). Each design was implemented in 3 pixel sizes - 100, 200, and 300 $\mu m$, allowing systematic investigation of finite-size effects.

Table 1 summarizes the number of unit-cell repetitions within a pixel for the different period lengths and pixel sizes. Figures S1 and S2 present the simulated and experimentally measured absorption spectra for varying periods at fixed radii of R = 0.9$\mu m$ (Fig. 1) and R = 1.15$\mu m$ (Fig. S2), respectively. For the smallest period (P = 2.4$\mu m$), a 100$\mu m$ pixel contains 41 unit cells, whereas a 300$\mu m$ pixel contains 125 unit cells. As shown in Fig. S1, the 300$\mu m$ pixel exhibits excellent agreement with the infinite-periodic simulations, while smaller pixels display a uniform reduction in absorption. As the period increases, the number of resonant elements per pixel decreases, leading to reduced absorption even for the 300$\mu m$ pixels. This effect scales similarly for smaller pixel sizes. From these observations, we conclude that at least ~100 unit-cell repetitions are required to approach the ideal periodic regime. These measurements establish the unit-cell period as an effective degree of freedom for tuning the absorption amplitude in finite metasurface arrays and highlight a fundamental trade-off between total emission and pixel size. Based on this trade-off, the final device design employs pixels with a size of 200 μm. Using 300$\mu m$ pixels, peak relative emissions of 44.3% in the LWIR and 33.7% in the MWIR are achieved.

*Table 1 – The number of fabricated periods for a given pixel array size in a range of relevant periodicities.*

| Pixel array size | Period length ($\mu m$) | Number of periods |
| --- | --- | --- |
| 100 | 2.4-5.2 | 41-19 |
| 200 | 2.4-5.2 | 83-38 |
| 300 | 2.4-5.2 | 125-57 |


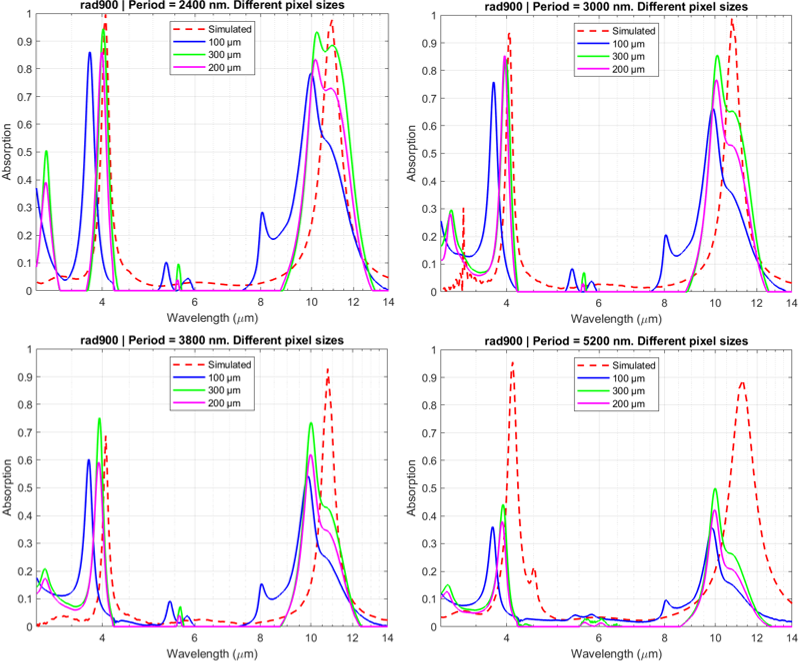


**Fig.S1 – Simulated and experimentally measured absorption spectra** for 4 different periods at fixed radii of R = 0.9$\mu m$ and for 3 different array sizes. The absorption reduced uniformly as the array gets smaller.


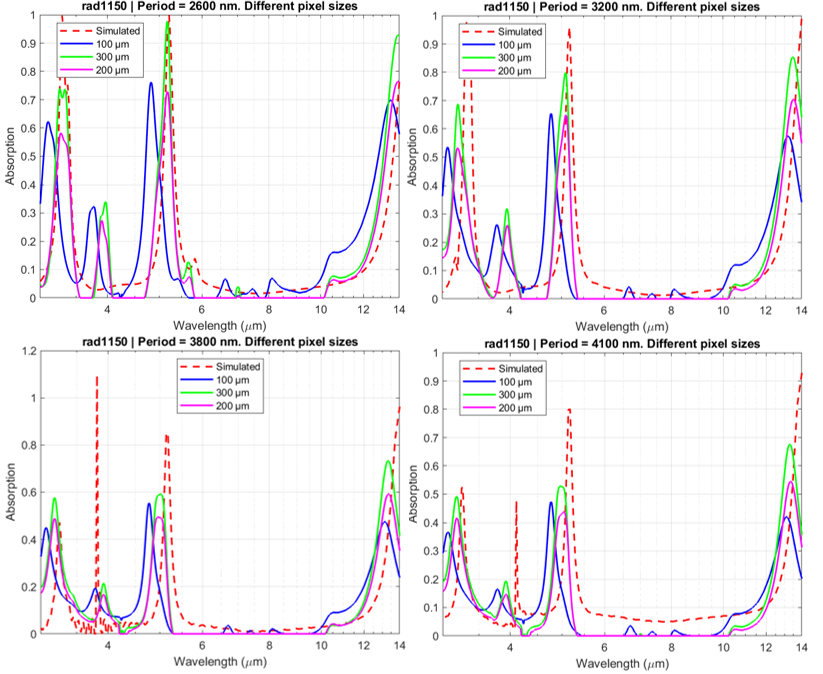


**Fig. S2 – Simulated and experimentally measured absorption spectra** for 4 different periods at fixed radii of R = 1.15$\mu m$ and for 3 different array sizes. The absorption reduced uniformly as the array gets smaller.

**2 - SUPPLEMENTARY NOTE: 130 Thermal Pixels - Fabrication and Measurements**

In the main manuscript we showed the relative MWIR/LWIR emission of 130 different periodic structures, with unit cell radius of $0.7\leq R\leq1.2\mu m$ and periods of $2\left( R+0.1\mu m \right)\leq P\leq5\mu m$. These values were sampled with 2 thermal cameras (MWIR and LWIR) and the gray-scale values were normalized using a blackbody set to the same temperature as the structures. Fig.S3 shows FTIR measurements and FDTD simulations of 49 structures used in the main manuscript. The main difference between the simulated values and the measured values is noticeable in high periods. As the period increases, we see a decrease in total absorption (see Supplementary Note 1). In our devices, we kept a fixed pixel size of $200\mu m$. Since pixel size is fixed, structures with higher period values have less unit cells, while in the FDTD simulations we assumed symmetrical boundary conditions.

The results in figures 2 to 4 in the main manuscript are of devices fabricated with E-beam lithography. The density of the disks as well as their sizes vary significantly in our devices. For small periods, the distance between adjacent disks is 200$\mu$m which is less than 10% for larger radii. For higher periods, the disks are far apart. Fig.S4 shows SEM images of disks of the same radius but different periods. The different proximities in our devices do not seem to affect fabrication.

The dimensions of our unit cells (arrays of disks with minimal diameter of $1.4\mu m$ and minimal distance of $200\mu m$ between them are big enough) are suitable for photolithography fabrication. Using photolithography enables quick repetitive writing of big arrays. In the main manuscript the fabrication was done using E-beam lithography, but we were also able to show the capability to fabricate such features (disks with the appropriate radius and periods) by photolithography. Fig.S5 shows a comparison between disks fabricated in E-beam and disks of the same properties fabricated in photolithography.

*
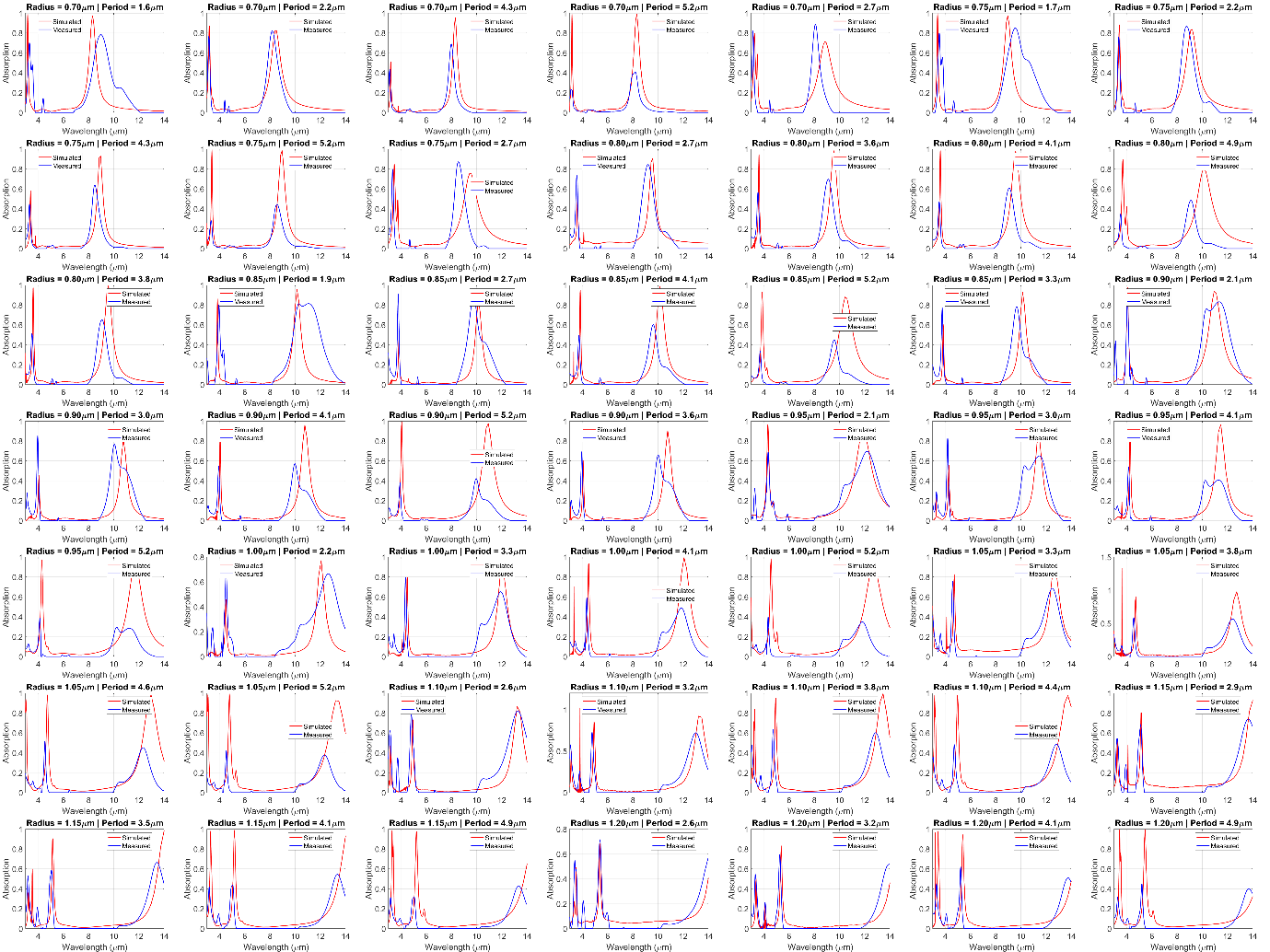
*

Fig.S3 – **FTIR measurements Vs. FDTD simulation results for 49 of the 130 measured structures.**


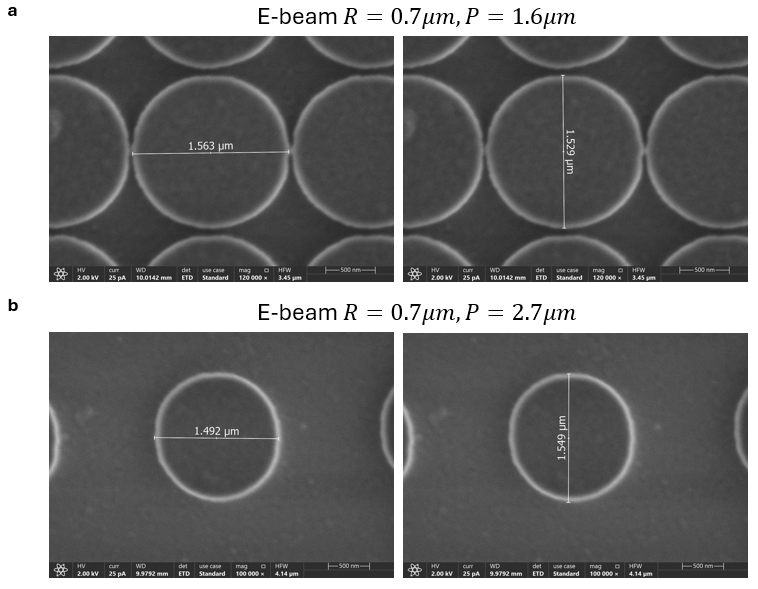


Fig.S4: **SEM images for fabricated disks with the same radius but different periods. a.** $R=0.7\mu m, P=1.6\mu m$. Disk’s diameter in x-axis is $1.563\mu m$ and in y-axis is $1.529\mu m$. **b.** $R=0.7\mu m, P=2.7\mu m$. Disk’s diameter in x-axis is $1.492\mu m$ and in y-axis is $1.549\mu m$. The same writing parameters were used for both cases, and the proximity of features doesn’t seem to affect the process. There are some variations between disks and some ellipticity.


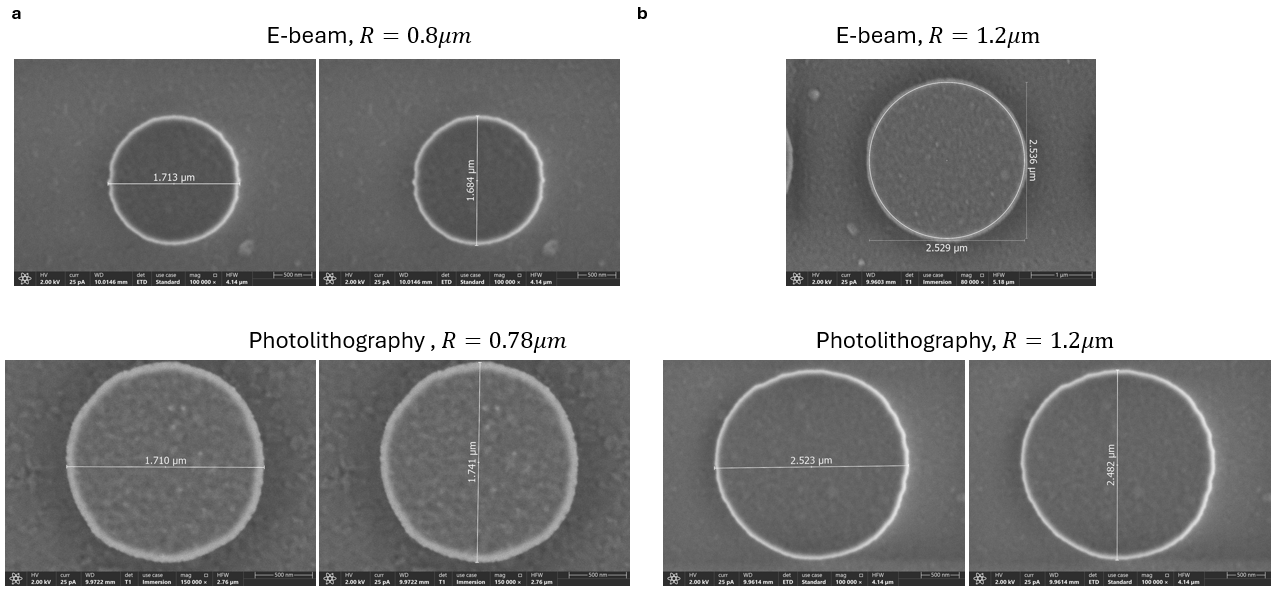


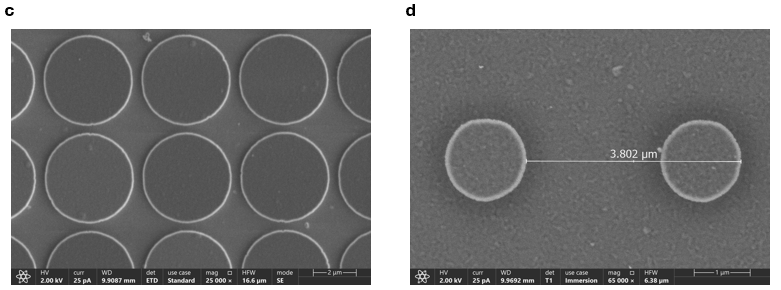


Fig.S5: **Comparison between disks fabricated in E-beam and photolithography. a.** E-beam fabricated disk designed with $R=0.8\mu m$. A photolithography mask with $R=0.78\mu m$ yielded similar results with similar ellipticity. **b.** A comparison of E-beam and photolithography for $R=1.2\mu m$. **c-d.** Photolithography fabrications of disks in close proximity (c, ~400nm) and in long periodicity (d).

**3 — SUPPLEMENTARY NOTE: Temperature Stability and Fabrication Tolerances**

A robust dual-band thermal platform must remain stable under temperature variation and realistic fabrication tolerances. In this note, we analyze the geometric fabrication tolerances,
and the induced effect on experimental results in comparison with simulations. We next discuss two mechanisms for temperature instability - temperature-dependent material dispersion and radiometric spectral redistribution due to Wien’s displacement. Finally, we model the device's optical stability under macroscopic thermal gradients and non-ideal heating conditions. We also provide the thermal images at elevated temperatures up to 112 °C, to complete the data analysis as shown in fig4c-d in the main manuscript.

**3.1 Fabrication Tolerances and Geometric Sensitivity**

The metasurface consists of a metal–insulator–metal (MIM) cavity comprising:

- 100 nm Al ground plane (optically opaque mirror),
- d=215 nm amorphous silicon (a-Si) spacer, and
- t=50 nm patterned Al disks of radius *R*.

The effect of the geometric parameters on the measured resonances can be predicted rigorously by modeling the MIM unit cell as an RLC circuit. The absorption occurs when the external magnetic field induces anti-parallel currents in the disk and the ground plane, forming a closed current loop, with a magnetic inductance of $L_{m}\propto d$. Additional inductance is the kinetic inductance, $L_{k}$ due to the inertia of the electrons in the thin disks $(L_{k}\propto1/Rt )$. The total inductance, $L_{total}$, is the sum of $L_{m}$ and $L_{k}$. The disk and the ground plane form a parallel-plate capacitor, with a capacitance $C_{m}\propto R^{2}/D$. The resonance frequency follows $\omega_{res} = 1/\sqrt{L_{total}C_{m}}$. By substituting the terms above, we can indeed show that $\omega_{res}\propto1/R$, as we demonstrated in Fig.1 of the main manuscript. Moreover, the spacer thickness (d) simultaneously dictates the gap capacitance ($C_{m}\propto1/d$) and the magnetic inductance ($L_{m}\propto d$). Because the total inductance also includes the kinetic inductance, the product $L_{total}C_{m}$ is not constant; rather, as d increases, the decrease in capacitance dominates, and shall lead to a higher resonance frequency (blue shift). The resonance frequency depends on t only via the kinetic inductance. Increasing the Al thickness reduces $L_{k}$ , which reduces the total circuit inductance $L_{total}$ and therefore increases $\omega_{res}$ (blue shift).

With this RLC equivalent model, we turn to analyzing the effect of our Fabrication Tolerances on the spectral response**.**

The bottom Al layer functions solely as a reflective mirror. Once its thickness exceeds ~50 nm (according to FDTD simulations), further increase does not significantly affect spectral response; we therefore used 100 nm to ensure full optical opacity.

The a-Si spacer thickness controls the cavity resonance and is therefore a critical design parameter. To quantify sensitivity, we simulated absorption spectra for spacer thicknesses of 200, 215, 240, and 270 nm under two representative geometries - the nonlinear coupling regime (*R* = 0.7 μm, *P* = 1.6 μm, Fig.6Sa) and the linear regime (*R* = 0.8 μm, *P* = 3.8 μm, Fig.6Sb) as further discussed in Supplementary Note 4. For both cases, thickness modifies resonance depth while in the linear case there’s also a spectral shift. The chosen value of 215 nm provides balanced MWIR and LWIR emissivity levels and was selected as a practical design compromise rather than a strict global optimum.

Ellipsometry measurements indicate a fabrication tolerance of ±~7 nm after calibration (see methods), with a systematic upward shift yielding measured thicknesses between 220–235 nm. From the simulated thickness sweep (as shown in Fig.S6a-b) , this variation induces up to ~5% change in peak emissivity and a small resonance shift. This resonant shift is consistent with the measured deviations between FDTD simulations and FTIR spectra in Fig. 1 of the main manuscript, where experimental resonances exhibit a slight blue shift compared to simulations. This observed discrepancy can therefore be attributed primarily to realistic fabrication tolerances rather than modelling inaccuracies (in the FTIR-measured results, we also see inhomogeneous broadening of the resonances, which can be attributed to slight variations in spacer thickness, as discussed here, but more importantly, variations in disk diameter).

Figures S6(c–d) present simulated absorption spectra for variations in the top Al disk thickness. The fabrication tolerance on this thickness is less than 10%. The nonlinear coupling regime (Fig.S6c), which is more sensitive to this perturbation, exhibits only minor changes in resonance amplitude and linewidth under these thickness variations. The linear regime (FigS6.d) is stable over a wide range of thicknesses.


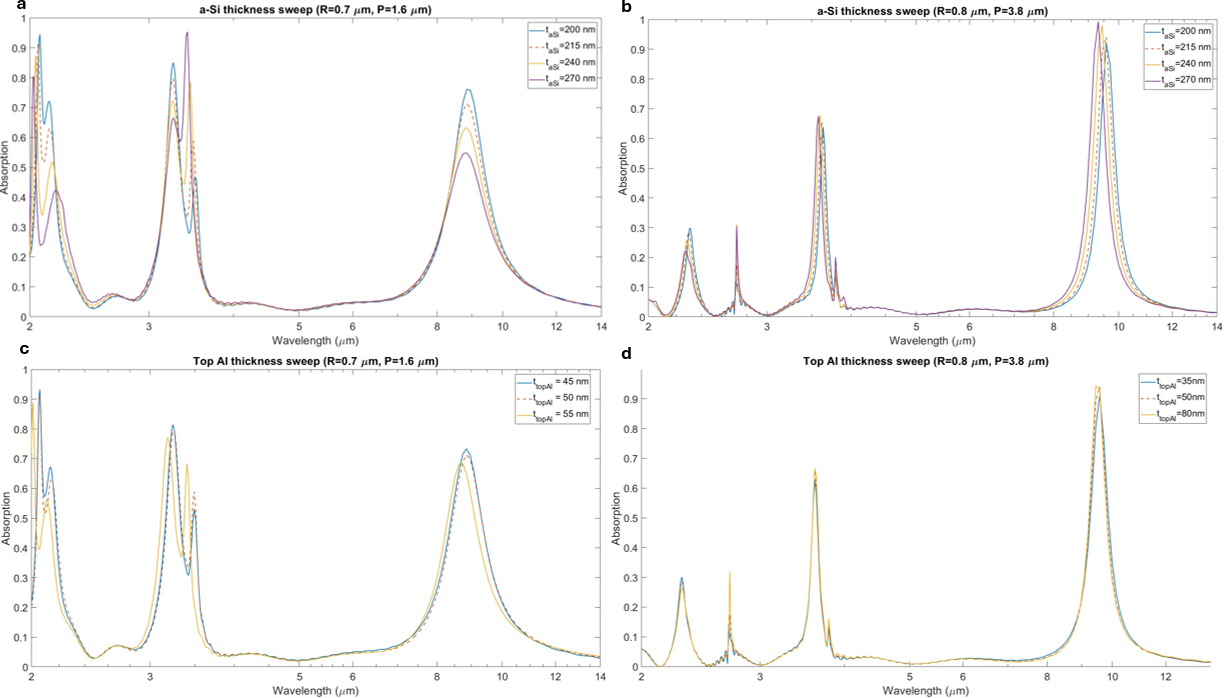


***Fig. S6 | Sensitivity of spectral response to changes in layer thickness. a-b.*** *Simulated absorption spectra for changes in the a-Si layer thickness in the nonlinear coupling regime (a) and in the linear regime (b). The selected value (215 nm) provides balanced MWIR and LWIR response.* ***c-d.*** *Simulated absorption spectra for changes in the top Al layer thickness in the nonlinear coupling regime (c) and in the linear regime (d).*

**3.2 Temperature-Dependent Material Dispersion and Optical Stability**

The platform does not rely on phase transitions or thermally induced resonance switching. The optical response is governed by cavity geometry and material permittivity.

For amorphous silicon in the mid-infrared, reported thermo-optic coefficients ($\frac{\boldsymbol{dn}}{\boldsymbol{dT}}\boldsymbol{)}$ are typically in the range of $1-3\cdot{10}^{-4}K^{-1}$. Using a conservative upper value, the change in refractive index between 37 °C and 120 °C (ΔT ≈ 83 K) is $\Delta n\approx2.5\times{10}^{-2}$. Given a mid-IR refractive index of $n\approx3.4$, this corresponds to a relative index change ($\frac{\Delta n}{n})$ of $\sim0.7\%$. The equivalent thickness variation induced by thermo-optic dispersion is therefore:

$$\Delta t_{eff}=t_{aSi}\frac{\Delta n}{n}\approx1.5nm$$

for a dielectric layer of 215nm, according to our platform design.

This value is significantly smaller than the measured fabrication tolerance as discussed earlier. Even if extrapolated to 300 °C (ΔT ≈ 260 K relative to room temperature), the corresponding effective thickness variation remains on the order of ~4–5 nm, still below fabrication-induced variations. Therefore, temperature-induced dispersion effects are smaller than geometric tolerances, which are handled in FigS6 and the above discussion.

**3.3 Radiometric Redistribution with Temperature (Wien Shift Effects)**

In addition to structural and material stability, temperature affects the emitted spectrum through blackbody redistribution.

According to Wien’s displacement law:

$$\lambda_{\mathrm{peak}}\propto\frac{1}{T}$$

As temperature increases, spectral weight shifts toward shorter wavelengths. Since the device response is determined by overlap between cavity resonances and blackbody emission, this shift modifies relative MWIR and LWIR emission slightly.

Fig. S7 shows simulated relative emission at 250 K, 300 K, and 400 K. The maximum variation between 300 K and 400 K remains below ~4%, confirming weak temperature dependence of the normalized emissivity metric.

Thus, radiometric spectral redistribution does not significantly degrade dual-band selectivity across practical temperature ranges.


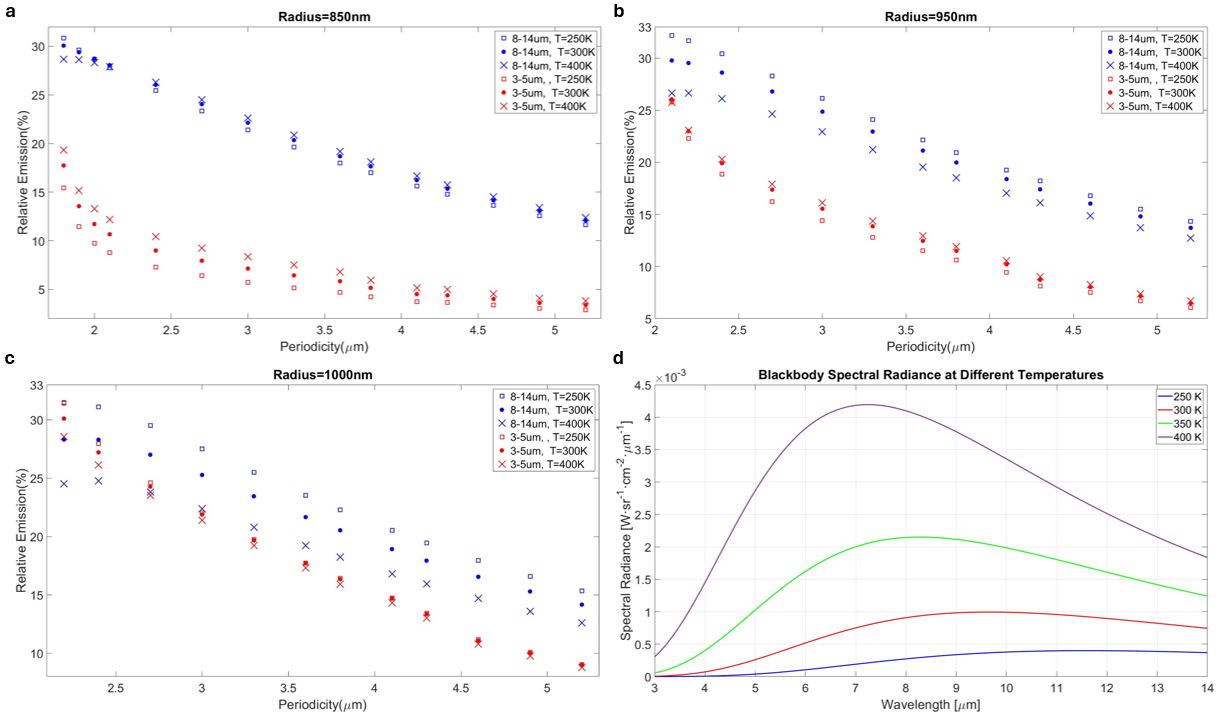


Fig.S7: **Relative thermal emission in MWIR (**$\boldsymbol{3-5}\boldsymbol{\mu m}\boldsymbol{)}$ **and LWIR (**$\boldsymbol{8-14}\boldsymbol{\mu m}$**) at different temperatures. a-c.** The relative thermal emission for 250,300 and 400K is shown for all structures with the fixed radius stated in graph’s title. The results are based on FDTD simulations and Planck’s law (eq.1) for the given temperatures. The maximal measured difference in relative emission for the same structure in 300K compared to 400K is 4%. **d.** The blackbody radiation at different temperatures of 250,300,350 and 400K. The total emission of each thermal structure in each thermal band, for a given temperature, is the integral of this function times the spectral emissivity function.

**3.4 Stability under Macroscopic Thermal Gradients and Non-Ideal Heating**

Applications such as thermal camouflage and dynamic thermal management are often deployed in dynamic environments characterized by non-uniform temperature distributions, imperfect thermal contact, and localized cooling. To address the stability of our device under such non-ideal macroscopic conditions, we simulated the apparent radiant exitance of the encoded correlated dual-band image across a 133x133 pixel array. We modelled the spatial temperature distribution under four conditions: an ideal uniform heating at 70°C (Fig.S8a), a severe non-ideal environmental cooling scenario represented by a 20°C Gaussian temperature decay from the center to the edges (Fig.S8b), the same Gaussian decay with the addition of a $1^{\circ}c$ random pixel-to-pixel temperature deviation to simulate micro-scale thermal contact resistance (Fig.S8c), and extreme thermal noise with a $5^{\circ}c$ random deviation(Fig.S8d). As shown in Fig. S8, while the absolute radiant exitance scales with the local temperature, the emissivity-encoded image remains clearly resolvable even under massive macroscopic thermal gradients and severe local noise.


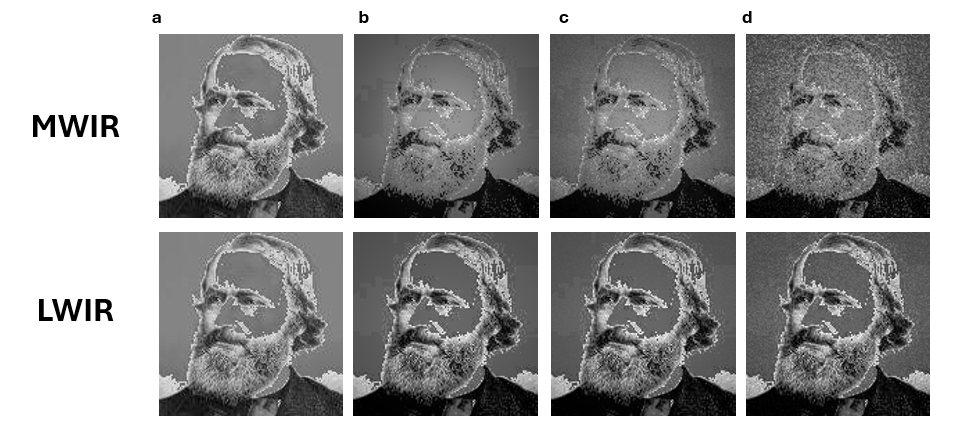


***Fig. S8 | Simulated thermal image stability under non-ideal heating and thermal gradients.*** *Simulated apparent radiant exitance for the encoded 133x133 pixel image in the MWIR and LWIR bands under various temperature distributions.* ***(a)*** *Ideal uniform heating at 70°C.* ***(b)*** *Macroscopic thermal gradient represented by a 20°C Gaussian decay from the center (70°C) to the edges (50°C), simulating non-ideal thermal contact and edge cooling.* ***(c)*** *The same 20°C Gaussian decay with an added* $1^{\circ}c$ *random temperature noise per pixel.* ***(d)*** *The 20°C Gaussian decay with an extreme* $5^{\circ}c$ *random temperature noise. The encoded pattern remains highly visible across all non-ideal conditions, demonstrating the robustness of structural emissivity encoding.*

**3.5 Extended Experimental Validation (37–112 °C)**

Representative MWIR and LWIR thermal images are shown in Fig. 4b of the main manuscript, while the quantitative analysis presented in Fig. 4d includes additional temperature points not explicitly displayed there. For completeness, the full set of thermal images acquired between 37 °C and 112 °C is provided in Fig. S9.


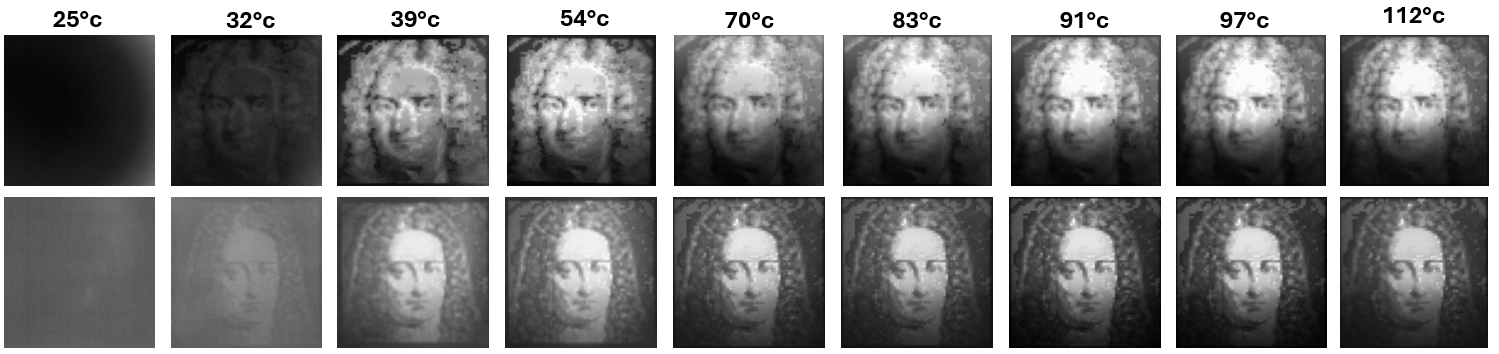


***Fig. S9 | Complete experimental thermal image set from 37 °C to 112 °C.*** *Full MWIR (3–5 μm) and LWIR (8–14 μm) thermal images acquired across the entire experimental temperature range. The spatial patterns remain stable and grayscale contrast scales monotonically with temperature, confirming robustness of dual-band emissivity control over the full measured range.* *For temperatures above 54°C, MWIR images were acquired using a neutral density filter (OD 1.0) to prevent detector saturation.*

**4 - SUPPLEMENTARY NOTE: Quantitative Analysis of Mode Orthogonality and Control Stability**

To rigorously address the potential for pattern coupling and nonlinear interference between the MWIR and LWIR bands, we performed a multi-level analysis linking the microscopic near-field interactions to the macroscopic control stability.

**4.1 Physical Mechanism: Local Field Hybridization**

The independence of the MWIR and LWIR resonances relies on the localization of electromagnetic modes within the individual MIM unit cells. When the unit-cell period (P) is sufficiently large relative to the resonator radius (R), the localized surface plasmon resonances (LSPRs) remain confined, treating each unit cell as an isolated cavity. However, as the period decreases and approaches the physical limit of the resonator size $(P\approx2R$), the evanescent fields of adjacent resonators begin to overlap, leading to mode hybridization (nonlinear coupling).


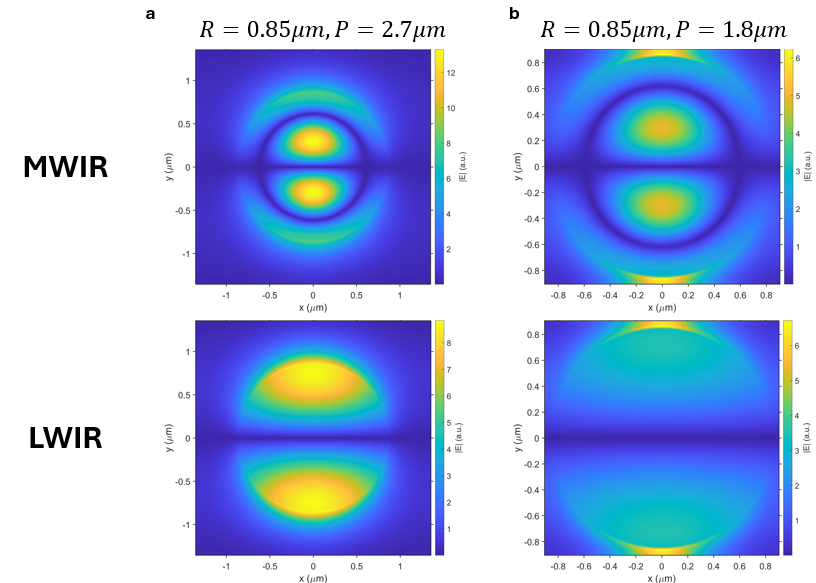


***Fig. S10 | Near-field distributions and mode hybridization.*** ***(a)*** *FDTD simulation of the electric field magnitude |E| at the MWIR (up) and LWIR (down) resonances for a decoupled design (*$R=0.85\mu m P=2.7\mu m$*). The mode is tightly localized to the MIM resonator structure.* ***(b)*** *Electric field magnitude for a coupled design (*$R=0.85\mu m P=1.8\mu m$*), near the geometric singularity. Significant field overlap between adjacent unit cells is visible, indicative of the onset of nonlinear pattern coupling.*

Figure S10 illustrates this transition using FDTD simulations of the electric field distributions (|E|).

- **Decoupled Regime (Fig. S10a):** For a geometry in our independent design palette
  ($R=0.85\mu m P=2.7\mu m$), the fields are strongly confined to the nano-disk edges with negligible near-field overlap between neighbors.
- **Hybridized Regime (Fig. S10b):** In the dense limit ($R=0.85\mu m P=1.8\mu m$), significant field leakage is observed in the gap between structures. This near-field coupling alters the resonant condition, creating a collective mode where independent control of the MWIR and LWIR bands is lost.

**4.2 Spectral Orthogonality and resonance anomalies**

To verify the geometric tunability in the decoupled regime, we analysed the experimental FTIR spectra. The data generally confirm that P acts as an amplitude control and R as a spectral tuner but also reveal irregularities that necessitate a robust stability analysis.


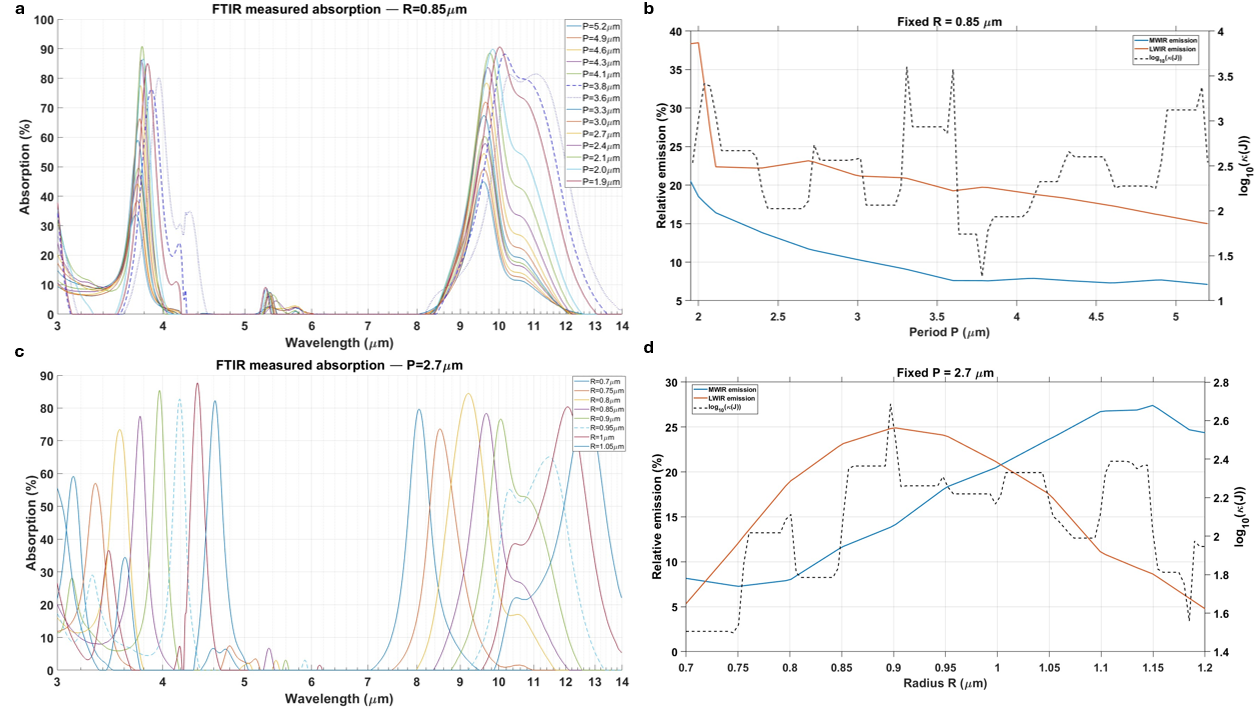


**Fig.S11 | Experimental verification of spectral orthogonality.** **(a)** Waterfall plot of emissivity (log-scale wavelength) for fixed radius ($R=0.85\mu m)$) and varying periods. Solid lines show the primary amplitude-scaling trend. Dashed lines ($P=3.6, 3.8\mu m$) highlight irregularities in the spectral trend. **(b)** Integrated emissivity values for the fixed R case, together with the corresponding Jacobian Condition Number (log-scale) for this scan, quantifying the control stability. **(c)** Waterfall plot for fixed period ($P=2.7\mu m$) and varying radii, showing spectral tuning. Increasing R causes a redshift in the resonance along with an uncorrelated change in the modulation depth. The dashed line ($R=0.95\mu m$) highlights an anomaly in the resonance shape. **(d)** Integrated emissivity values for the fixed P case, together with the corresponding Jacobian Condition Number (log-scale) for this scan, quantifying the control stability.

Varying the period for a fixed radius ($R=0.85\mu m$) mostly scales the emissivity magnitude confirming the role of *P* as an amplitude control (Fig.S11a). In the nonlinear zone ($P= 1.9\mu m$), due to the near-field coupling discussed earlier, there’s a spectral shift a slight change in the resonance shape. Irregularities are observed at specific lattice conditions
 ($P=3.6, 3.8\mu m$, dashed lines), where there’s a spectral shift that is even bigger than the shift caused by the nearfield coupling. Integrating over the spectral emissivity for the two regions (MWIR and LWIR; fig.S11b) approves the general trend, but also further emphasizes the mentioned anomalies. To quantify the impact of these irregularities on control reliability, we plot (dashed black line) the Jacobian Condition Number for this scan. The condition number serves as a local stability metric and is further discussed in 4.3.

Conversely, varying the radius for a fixed period ($P=2.7\mu m$) shifts the resonance positions across the bands (Fig. S11c). While the spectral tuning trend is consistent, the peak shapes and modulation depths vary uncorrelatedly. This shape decorrelation actually aids in decoupling, as the peak intensities of the two bands do not scale linearly with one another, preventing collinearity in the control variables. The $R=0.95\mu m$ design (dashed line) exhibits an anomaly in its LWIR response. Fig.S11d shows the integrated emissivity of every design in the MWIR and LWIR spectra, along with the Jacobian Condition Number for this scan (dashed).

These two degrees of freedom allow the construction of a unique mapping between geometry (R, P) and emissivity $(\epsilon_{MW}, \epsilon_{LW}).$

**4.3 Global Control Stability: Jacobian Sensitivity Analysis**

To quantify the limits of this independence and systematically exclude regions of nonlinear coupling or other irregularities, we performed a global sensitivity analysis across the entire experimental dataset (N=130 designs). We computed the Jacobian matrix J at every point in the design space:

$$J=\left[ \begin{matrix} \frac{\partial\epsilon_{MW}}{\partial P} & \frac{\partial\epsilon_{MW}}{\partial R} \\ \frac{\partial\epsilon_{LW}}{\partial P} & \frac{\partial\epsilon_{LW}}{\partial R} \end{matrix} \right]$$

The stability of the inverse problem (mapping emissivity targets back to geometry) is governed by the Condition Number, $\kappa\left( J \right)= \left| \left| J \right| \right|\cdot||J^{-1}||$. A low condition number indicates robust, independent control, while a high condition number indicates singularity and mode coupling.

Fig.S12a maps the $log_{10}(\kappa(J))$ across the parameter space. The analysis reveals a broad "stable zone" (blue/purple regions) where $log_{10}(\kappa) < 2.5$. The statistical distribution of the condition number across our design palette shows a mean of $log_{10}\left( \kappa\right)= 2.17$, indicating that for most cases, the mapping between geometry and emissivity remains well-conditioned and invertible for independent thermal encoding. The dashed line in Fig. S12b ($det\left( J \right)\approx0$) marks the transition to the hybridized regime identified in 4.1 and exposes other irregular zones in the parameter space. This boundary corresponds to the geometric limit where pattern coupling dominates.


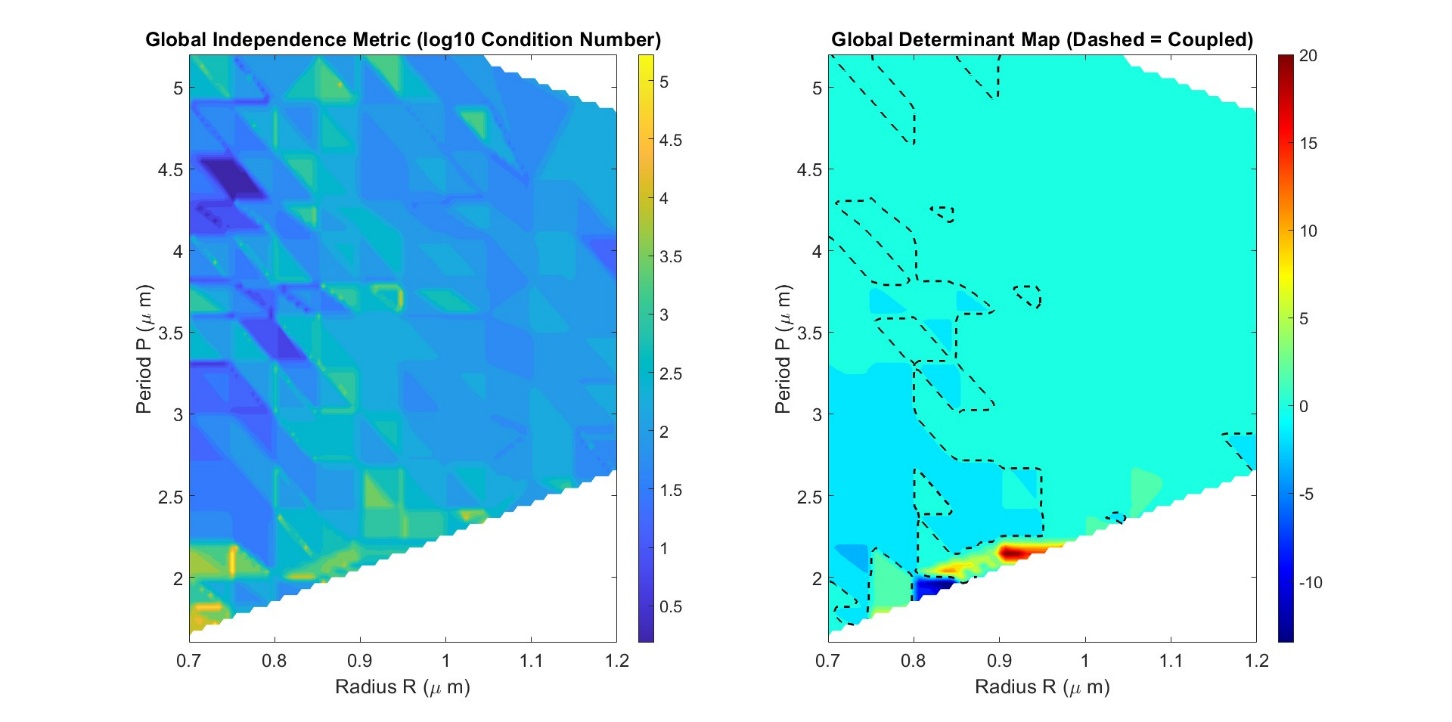


**Fig.S12 | Global Jacobian sensitivity analysis.** **(a)** Heatmap of $log_{10}(Condition Number)$ across the design space. Darker regions indicate the stable operational envelope where independent control is mathematically robust. This metric provides a quantitative tool to identify and filter out regions of strong coupling and spectral perturbations, ensuring that selected designs maintain dual-band orthogonality. **(b)** The Determinant map highlighting the singularity boundary ($det(J)=0$) where independent control is physically impossible.

**5 - SUPPLEMENTARY NOTE: Contextualization of Emissivity Dynamic Range in Camouflage and Encoding**

The measured average emissivity values reported in the manuscript (up to ~0.30 in MWIR and ~0.385 in LWIR) represent band-effective emission levels normalized to a blackbody reference at the same temperature. To interpret the practical impact of these values on thermal contrast, we use a standard first-order radiometric model for an opaque diffuse surface viewed in an environment at temperature $T_{\mathrm{bg}}$. The apparent signal is the sum of self-emission and reflected background radiation:

$$L_{\mathrm{app}}\propto\varepsilon\text{ }T_{\mathrm{obj}}^{4}+(1-\varepsilon)\text{ }T_{\mathrm{bg}}^{4}.$$

An equivalent “brightness temperature” (the temperature a camera would infer under a blackbody/graybody assumption) is then:

$$T_{b}=\left( \varepsilon\text{ }T_{\mathrm{obj}}^{4}+(1-\varepsilon)\text{ }T_{\mathrm{bg}}^{4} \right)^{1/4}.$$

For radiance units, we use the Stefan–Boltzmann proportionality as a broadband proxy:

$$M_{\mathrm{app}}\approx\sigma\left( \varepsilon\text{ }T_{\mathrm{obj}}^{4}+(1-\varepsilon)\text{ }T_{\mathrm{bg}}^{4} \right)[W/m^{2}],$$

where $\sigma$is the Stefan–Boltzmann constant. $M_{\mathrm{app}}$denotes the apparent radiant exitance

**5.1 Camouflage implications: “intermediate” emission is an advantage in complex scenes**

Many prior thermal-camouflage metasurfaces emphasize binary switching between a “high-emissivity” state (often approaching a blackbody in-band) and a “low-emissivity” state (near-zero, e.g., ~0.05–0.15). While this maximizes contrast control, it can be suboptimal in realistic scenes containing multiple objects and materials at different temperatures: driving emissivity too low can produce unnaturally cold-looking regions and strong background-reflection dominance (since reflectivity $\approx1-\varepsilon$), which can itself create detectability artifacts. In contrast, intermediate emissivity values support continuous “radiance matching” rather than “radiance removal.” The practical meaning is captured by the brightness-temperature mapping $T_{b}$: for a hot object, reducing $\varepsilon$compresses its apparent temperature toward the background.

In fig.S13, We plot $T_{b}$versus $T_{\mathrm{obj}}$ for $\varepsilon=0.1,0.2,0.3,0.4$ and background temperatures of $T_{\mathrm{bg}}=0,10,20,{30}^{\circ}C$. These curves quantify how the same physical object temperature can be made to appear significantly cooler (lower radiance) without forcing it to become “mirror-like cold.”

Background 20°C (fig.S13 c) is especially illustrative: at $T_{\mathrm{obj}}={120}^{\circ}C$, the apparent brightness temperature can get as high as 70°C and can be tuned across a broad range by modest emissivity variation. This supports camouflage in scenarios where an object must match a mid-level radiance consistent with nearby scene elements rather than being suppressed to the lowest possible radiance.

***Fig. S13 | Radiometric compression of apparent temperature for different background conditions.****Observed brightness temperature* $T_{b}$ *as a function of real object temperature* $T_{obj}$ *for 4 effective emissivity values (*$\varepsilon=0.1,0.2,0.3,0.4$*). The background temperature is assumed uniform and set to (a) 0 °C, (b) 10 °C, (c) 20 °C, and (d) 30 °C. The apparent temperature is calculated using* $T_{b}=\left( \varepsilon T_{obj}^{4}+(1-\varepsilon)T_{bg}^{4} \right)^{1/4}$*. The plots illustrate how moderate emissivity values compress the apparent thermal signature of hot objects toward the background temperature, enabling controlled radiometric matching rather than complete emission suppression.*


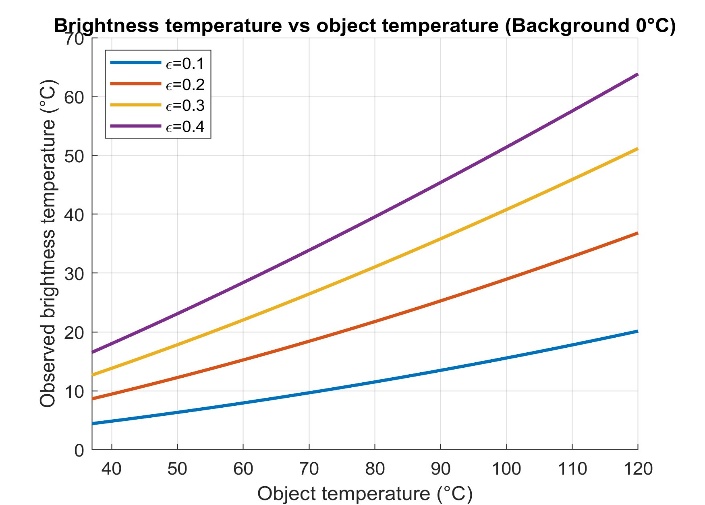

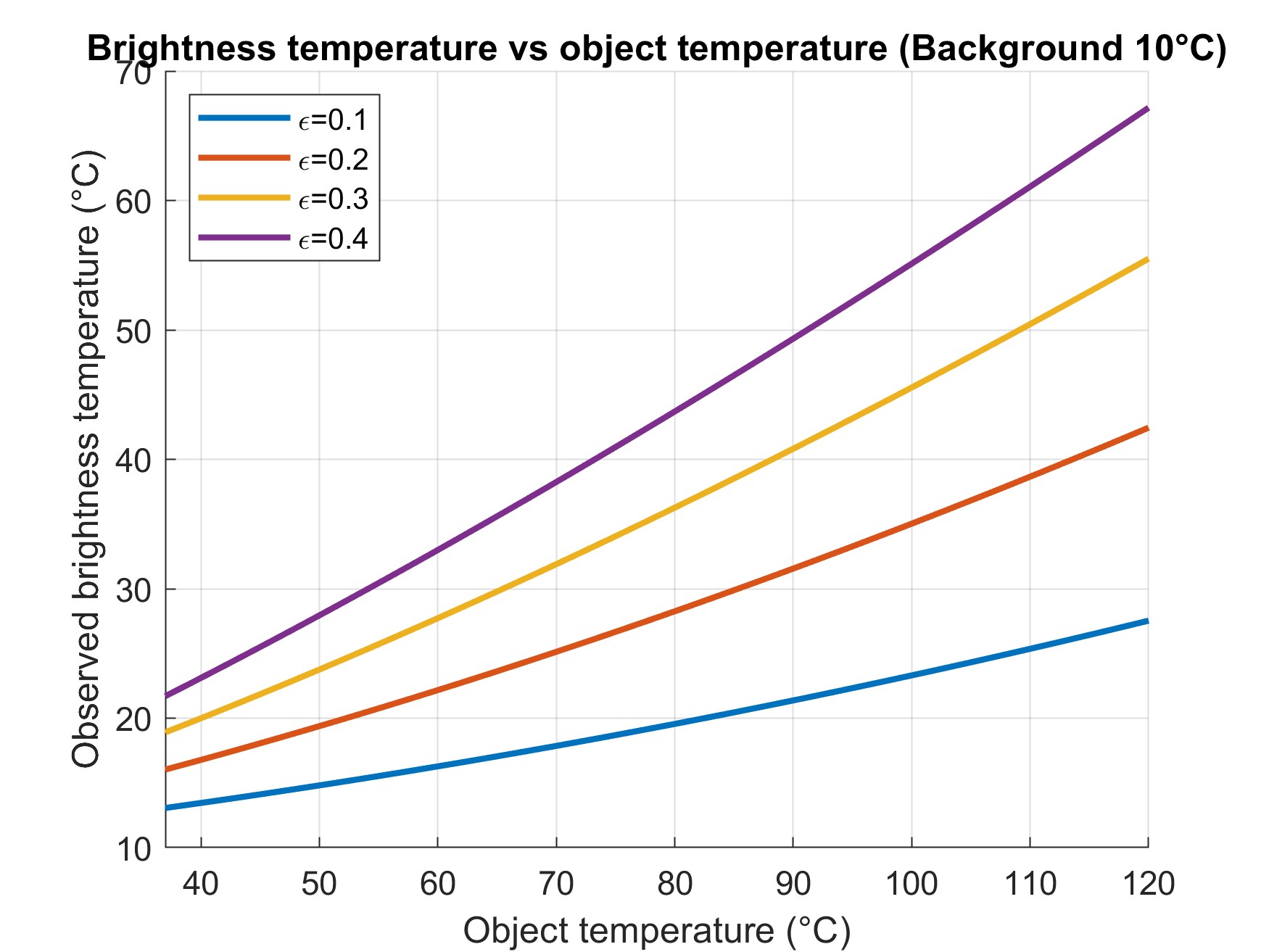

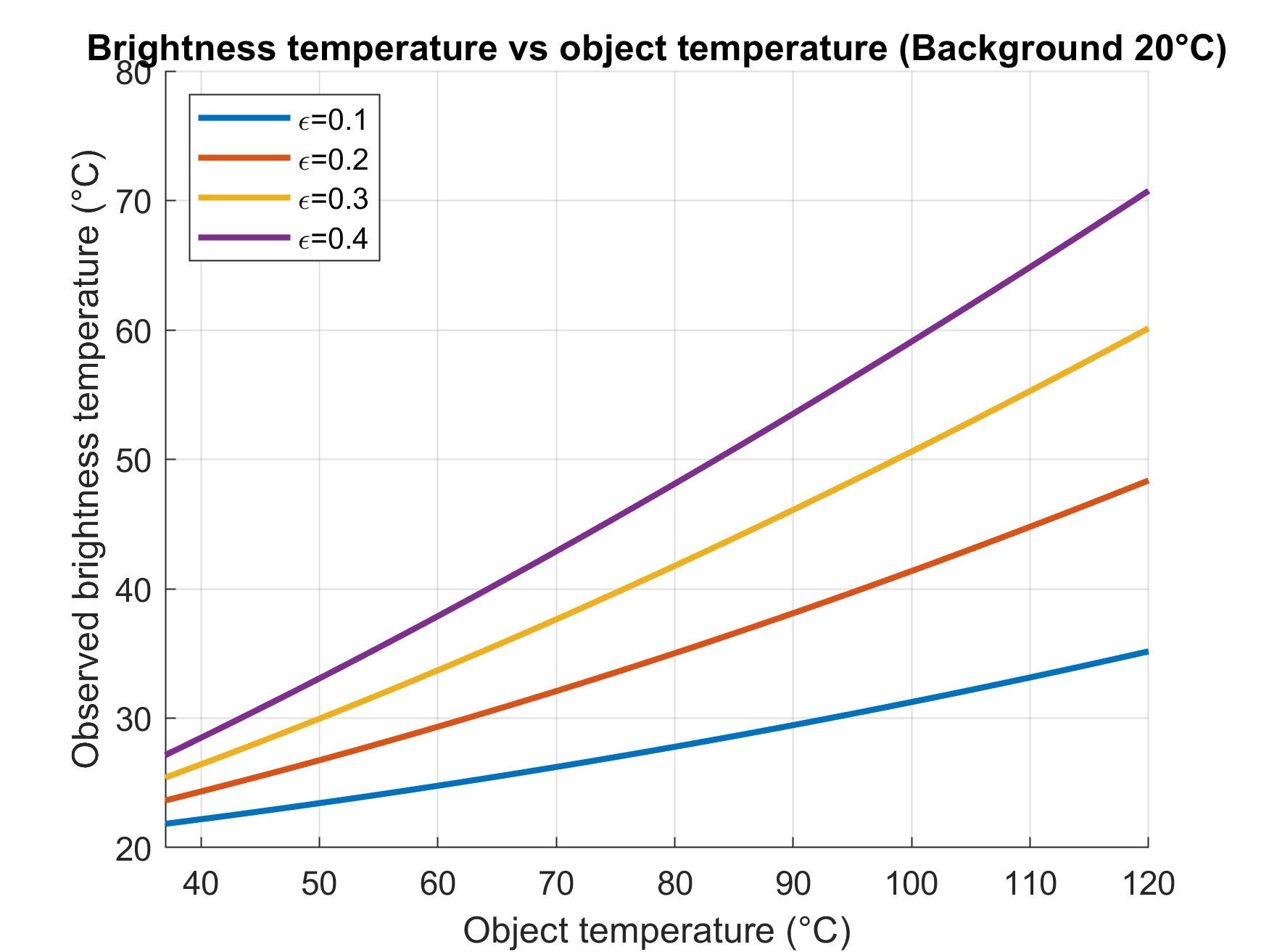

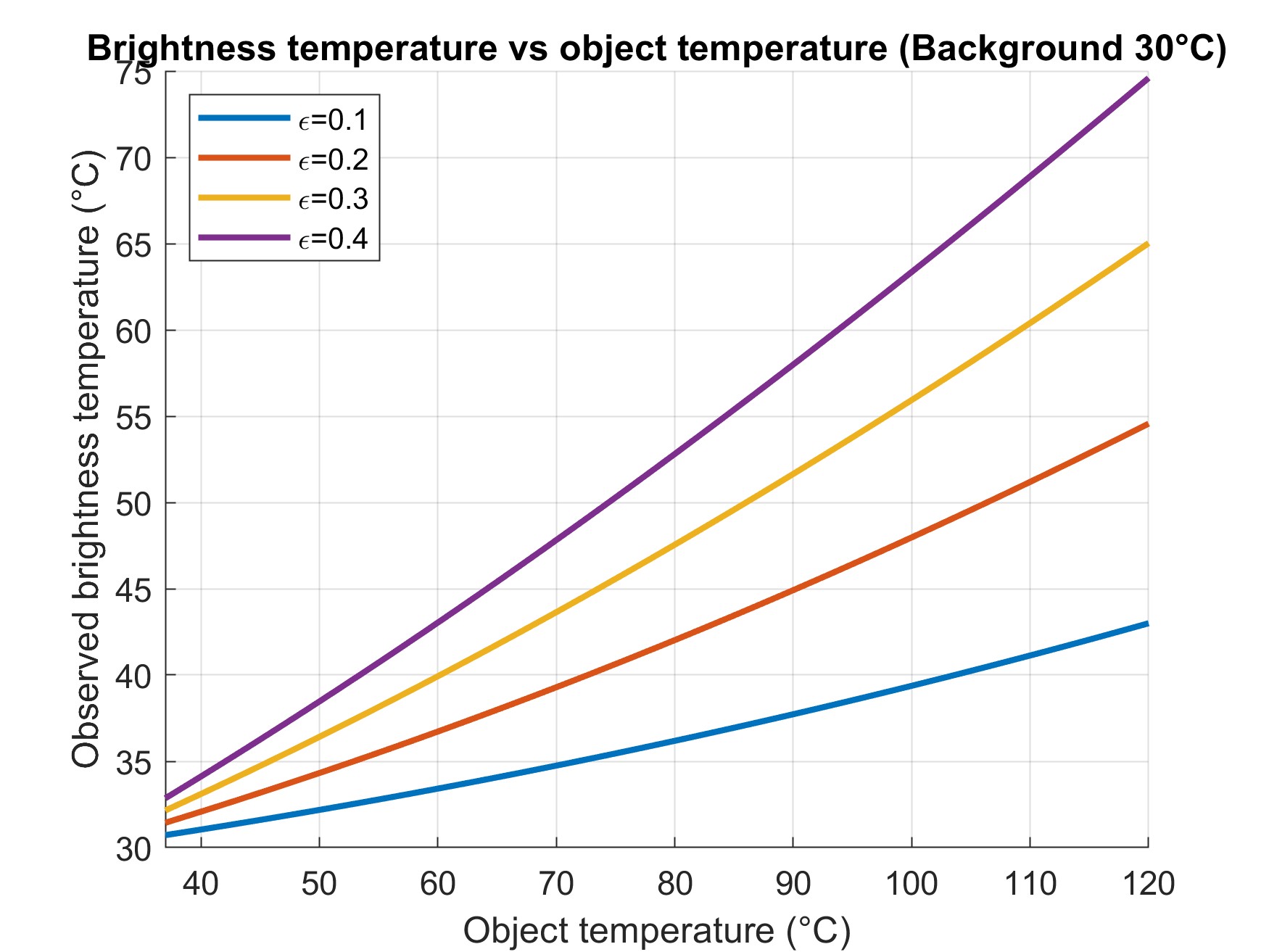


**a**

**b**

**c**

**d**

**5.2 Thermal encoding implications: multi-level emissivity yields large radiance contrast at practical temperatures**

For thermal encoding, the object temperature can be held uniform while information is encoded in spatial emissivity variations. In this regime, the relevant contrast is pixel-to-pixel variation in apparent radiant exitance:

$$\Delta M_{app}\approx\sigma\text{ }\Delta\varepsilon\text{ }(T_{obj}^{4}-T_{bg}^{4}).$$

Thus, even a moderate emissivity span (e.g., $0\to0.3$ for MWIR or $0\to0.385$ for LWIR) produces substantial contrast once $T_{obj}$ is elevated - exactly as observed experimentally when images become more prominent at higher temperatures.

In fig.S13 ,We plot $M_{app}$ in W/m² versus emissivity for $\varepsilon\in[0,0.4]$ at $T_{obj}=50,70,100,{120}^{\circ}C$ and backgrounds $T_{bg}=0,10,20,{30}^{\circ}C$. The plots show:

- Linearity vs ε at fixed temperature (expected from the model).
- Strong temperature leverage: the same emissivity step produces much larger radiance contrast at 100–120°C than at 50°C.
- Background temperature shifts the baseline radiance (the $(1-\varepsilon)T_{bg}^{4}$ term), but the slope with emissivity remains governed by $\sigma(T_{obj}^{4}-T_{bg}^{4})$.

**Fig. S13 | Apparent radiant exitance as a function of emissivity for thermal encoding.**
Apparent radiant exitance $M_{app}$(W·m⁻²) plotted versus effective emissivity ($\varepsilon\in[0,0.4]$) for uniformly heated objects at $T_{obj}=50,70,100,$and ${120}^{\circ}C$. The background temperature is assumed uniform and set to (a) 0 °C, (b) 10 °C, (c) 20 °C, and (d) 30 °C. The apparent radiant exitance is calculated using $M_{app}=\sigma\left( \varepsilon T_{obj}^{4}+(1-\varepsilon)T_{bg}^{4} \right).$ The plots illustrate the linear dependence of radiometric contrast on emissivity and the strong enhancement of encoding contrast with increasing object temperature due to the $T^{4}$scaling.


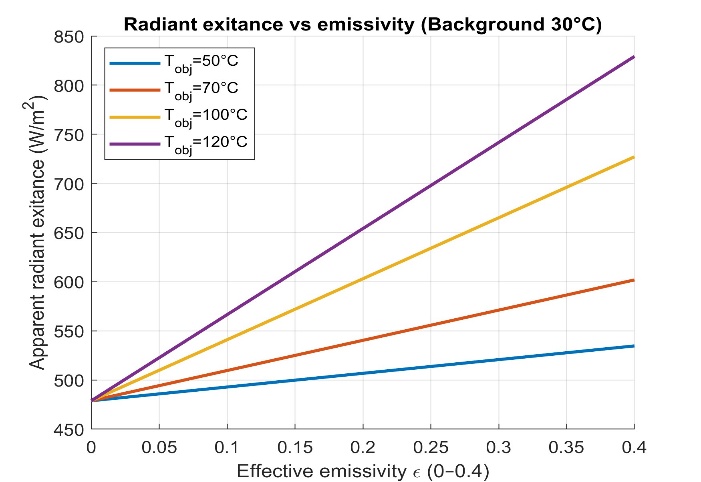

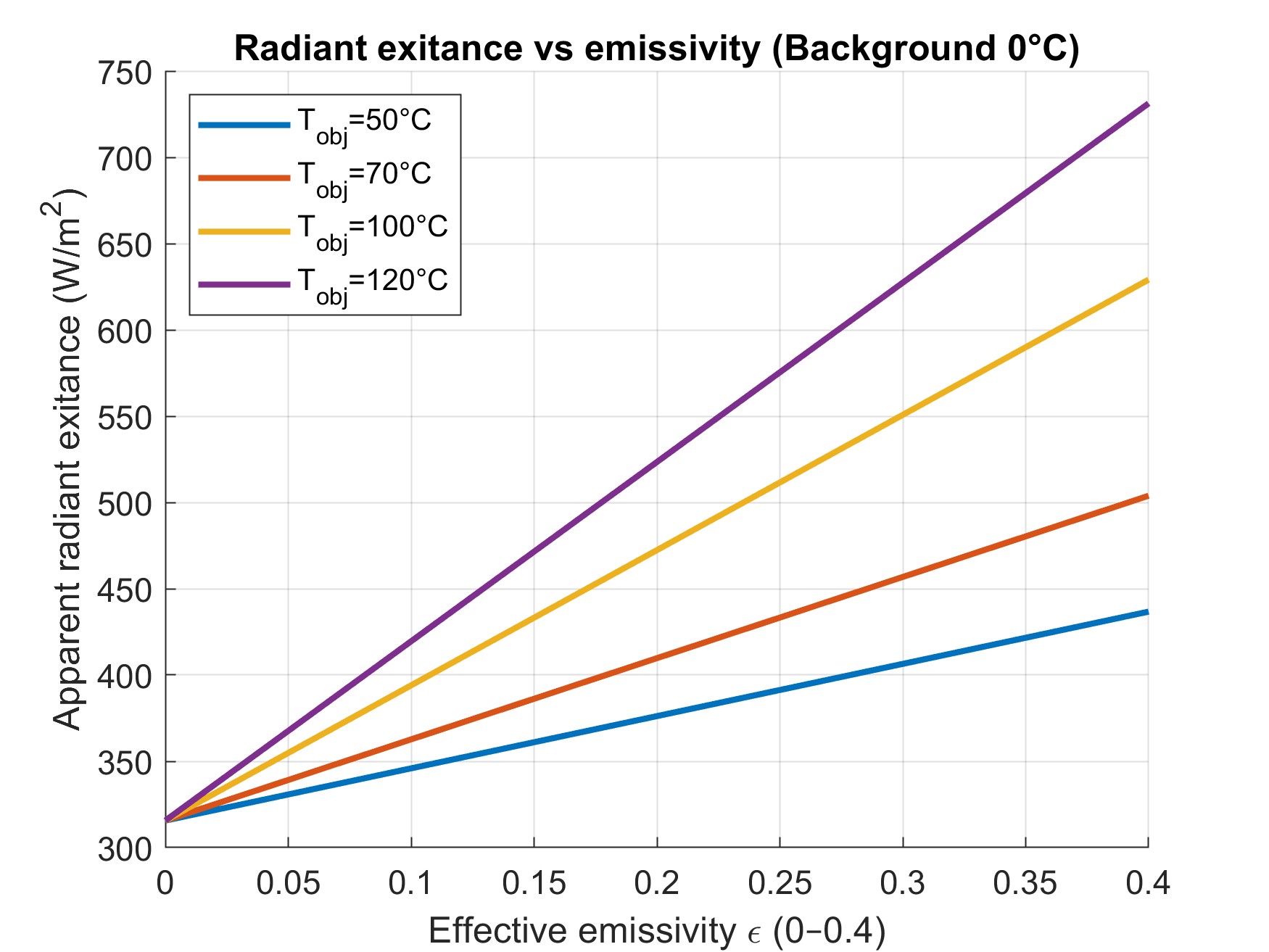

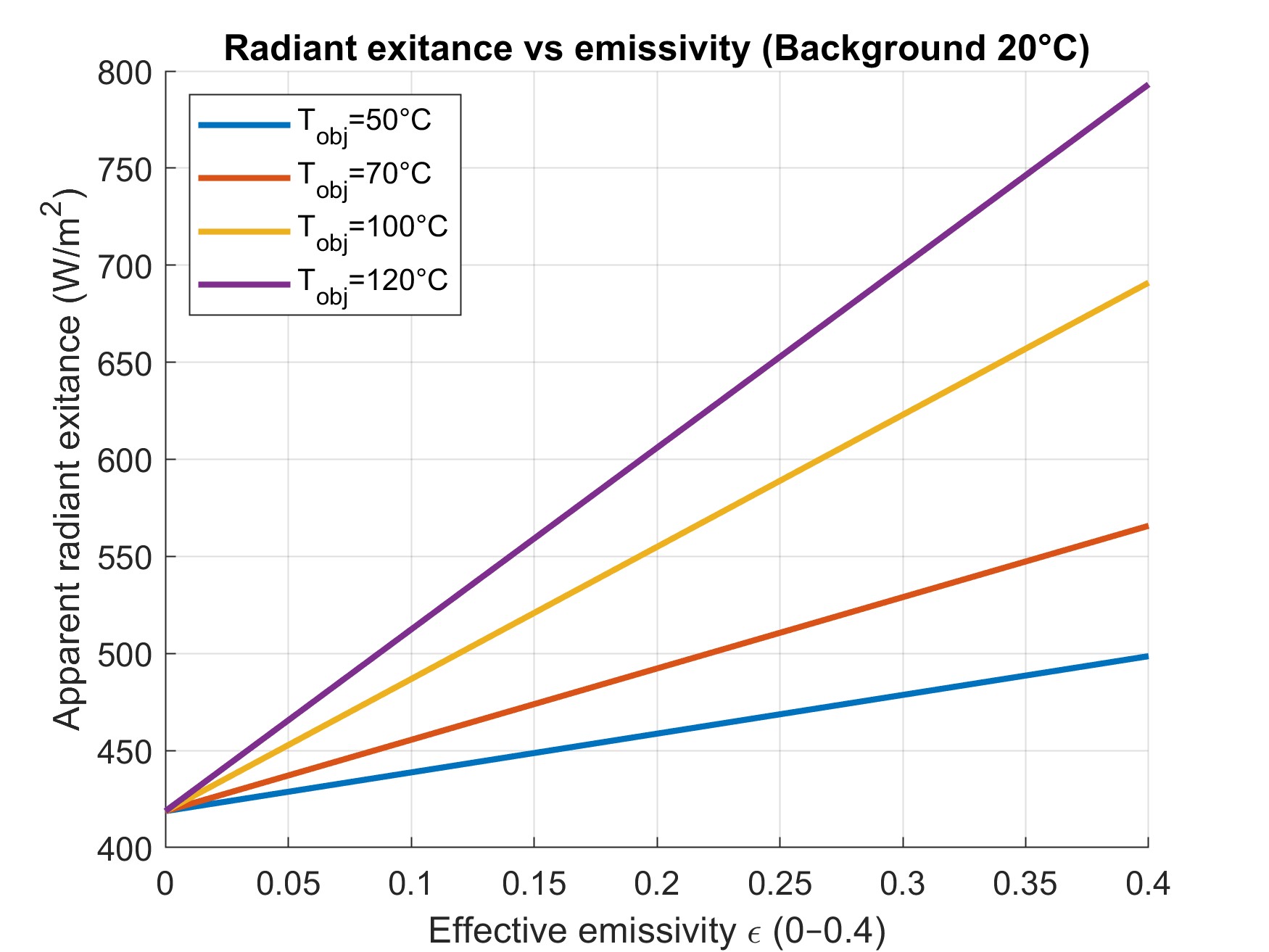

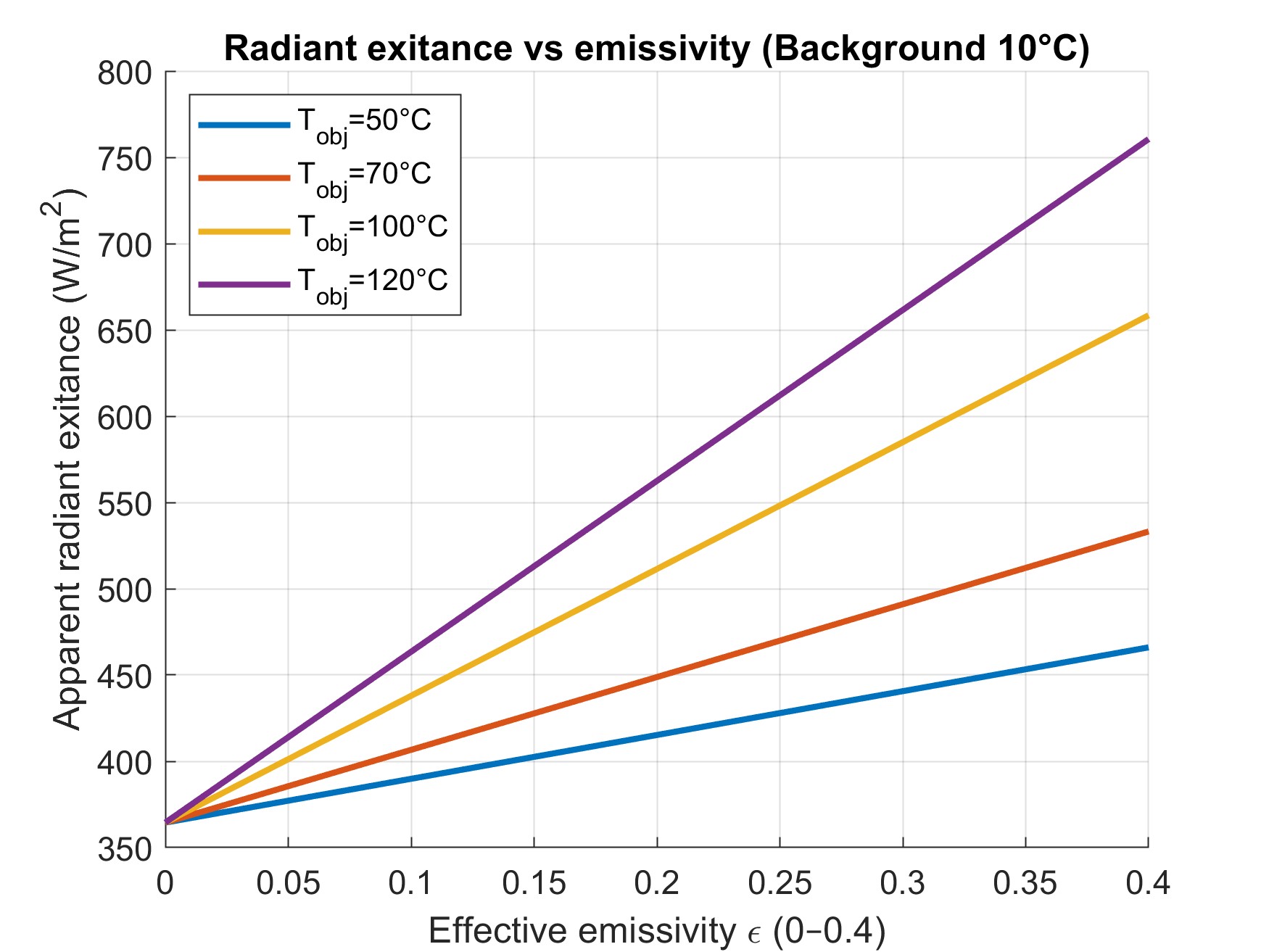


**a**

**b**

**c**

**d**
